# A Non-Equilibrium Thermodynamic Framework for Sequential Symmetry Breaking in Driven Complex Fluids

**DOI:** 10.3390/e28080910

**Published:** 2026-08-13

**Authors:** Antonio F. Miguel, Vinicius R. Pepe, Luiz A. O. Rocha

**Affiliations:** 1 Department of Physics, University of Evora, Rua Romao Ramalho 59, 7000-671 Evora, Portugal; 2 Complex Flow Systems Lab, Institute of Earth Sciences, Apart. 94, 7002-554 Evora, Portugal; viniciuspepe@gmail.com (V.R.P.); luizrocha@mecanica.ufrgs.br (L.A.O.R.); 3 PROMEC, University Federal of Rio Grande do Sul, Porto Alegre 91501-970, RS, Brazil

**Keywords:** active matter, complex fluids, non-equilibrium thermodynamics, symmetry breaking, tensorial hydrodynamics

## Abstract

The spontaneous emergence of macroscopic order in driven, far-from-equilibrium complex fluids lacks a generalized framework capable of bridging continuous and discrete symmetry-breaking transitions. In this study, we propose a non-equilibrium phenomenological framework that synthesizes irreversible thermodynamics, coupled Landau–de Gennes potential expansions, and active hydrodynamics. The formulation employs a single tensorial order parameter, a nonlinear state-dependent jamming mobility closure, and a generalized set of dimensionless groups to map the non-equilibrium phase space. The model predicts a sequential symmetry-breaking cascade and reproduces the emergence of polar heliconical smectic and antiferroelectric phases in driven liquid crystals, as well as the transition from isotropic active gases to macroscopic fluid flocks and active Wigner crystals in purely repulsive Janus colloids. Across these systems, a dimensionless active torque number acts as the principal bifurcation parameter, suggesting that their macroscopic structural transitions are governed by a common balance between thermodynamic and kinematic effects rather than by the details of their microscopic interactions.

## 1. Introduction

The spontaneous emergence of macroscopic order and spatiotemporal patterns from homogeneous, isotropic states stands as a defining characteristic of complex systems across physics, chemistry, and biology [1,2]. Driven by spontaneous symmetry breaking, these transitions govern phenomena spanning vastly disparate length and energy scales [3,4,5,6]. Despite this remarkable universality, a theoretical framework capable of bridging the continuous and discrete symmetry-breaking transitions observed across these diverse media has remained elusive, with most theoretical developments remaining confined to specific physical regimes or classes of materials.

The theoretical analysis of symmetry-breaking transitions has historically relied on effective nonlinear field equations that describe many-body systems through a minimal set of macroscopic variables. Foundational frameworks, such as the time-dependent Ginzburg–Landau theory for near-equilibrium phase transitions [7] and Turing’s seminal description of reaction-diffusion patterning [8], have proven effective in identifying generic mechanisms of scalar structure formation. However, highly directional, anisotropic media requires the implementation of tensorial order parameters that describe orientational rather than merely scalar order. Phenomena such as electrolubrication in polar liquid mixtures, where transverse electric fields induce significant concentration gradients and alter flow profiles [9], exemplify this requirement. The continuous hydrodynamics of such complex fluids were formalized through the Beris–Edwards thermodynamic framework [10], which established the coupling of structural tensor fields to Galilean-invariant macroscopic flows, providing a foundation for describing the relaxational dynamics of passive liquid crystals and polymeric fluids.

While classical approaches capture the relaxational dynamics of passive complex fluids near thermodynamic equilibrium, they require restructuring when applied to driven, active, or highly non-equilibrium media. In these systems, continuous energy injection at the microscopic scale shatters local thermodynamic stability, giving rise to phenomena that have no equilibrium analogues. The formulation of active gel theory [11] and broader hydrodynamic descriptions of active matter [12,13] demonstrated that non-equilibrium active stresses drive a wealth of novel phenomena. These range from the collective flocking of self-propelled agents [5,14] and the spontaneous, chiral symmetry-breaking self-propulsion of elliptical phoretic disks [15], to the chaotic emergence of topological defect turbulence in active nematics [16]. The challenge of deriving these macroscopic nonlinear field theories from underlying microscopic dynamics (a complicated study by the intrinsically non-equilibrium nature of active systems) has led to the widespread adoption of effective phenomenological models inferred from abstract symmetry considerations and bifurcation theory. However, these approaches often fail to capture the complex, non-linear interplay between advective kinematics, non-equilibrium driving forces, and locally coupled order parameters, limiting their predictive capacity and scope.

The panorama of phase transitions becomes increasingly complex when internal cross-couplings and dynamic boundaries determine the emergence of macroscopic order. Recent breakthroughs highlight that the symmetry of a pattern is frequently a dynamically selected property rather than a prescribed microscopic constraint. Spatiotemporal density fluctuations can induce local symmetry-breaking cascades, enabling the dynamic interconversion and coexistence of polar and nematic states [17]. This complex coupling is validated by the recent experimental discovery of spontaneous polar ordering within otherwise nematic fluid phases [18], revealing phase behavior that challenges conventional classifications. Additionally, the emergence of flocking in self-propelled colloidal systems with purely repulsive interactions and turn-away torques [19] demonstrates that macroscopic polar order can arise from microscopic interactions that do not implement alignment, challenging the assumption that polar order requires alignment interactions. These system-specific advances underscore the need for a framework that can describe symmetry-breaking phenomena across this diversity of driven, far-from-equilibrium systems.

Despite these extensive, system-specific advancements, bridging the breaking of continuous symmetries across such diverse ordered media requires a unifying thermodynamic perspective. While macroscopic geometries and scalar conduits have been analyzed through non-equilibrium thermodynamic imperatives [20,21], a complementary phenomenological framework that maps thermodynamic constraints onto the internal orientational and tensorial phase symmetries of complex fluids remains unavailable. Existing approaches, ranging from the equilibrium-centric Chen–Lubensky model for smectic liquid crystals to molecular field theories rooted in statistical mechanics [22], are each restricted to specific classes of phenomena, treat different order parameters on an unequal footing, and lack the capacity to describe active matter where continuous energy injection drives the system far from equilibrium. Recently, Miguel [23] proposed a field-theoretic framework that treats physical geometry as a dynamic state variable, represented by a time-dependent conductivity tensor, using a variational approach grounded in non-equilibrium thermodynamics to derive a general tensor evolution equation.

In this study, we propose a phenomenological framework that accounts for the spatiotemporal evolution of macroscopic order in driven, far-from-equilibrium complex fluids and soft matter systems. By synthesizing principles of non-equilibrium thermodynamics [21] with coupled Landau–de Gennes potential expansions [22], we derive a single tensorial evolution equation that links continuous and discrete symmetry-breaking transitions. The framework is grounded in a general convex dissipation potential that accommodates both linear and nonlinear flux–force relations, ensuring thermodynamic consistency across a broad range of driving conditions. Rather than relying on specific microscopic rules or ad-hoc phenomenological assemblies, our framework accommodates continuous spatial regularizations, emergent non-equilibrium active torques, and local symmetry-breaking cascades caused by the spatial fluctuations of a generalized scalar control parameter. Through dimensional analysis, we define generalized Deborah, Ericksen, Péclet, and dimensionless active torque numbers that map the complex non-equilibrium phase space onto a minimal set of independent dimensionless groups, eliminating arbitrary scaling conventions and providing a systematic framework for comparing disparate physical regimes. Finally, we show that this continuous field theory collapses into the distinct asymptotic regimes governing highly disparate phenomena, revealing that the complex geometric topologies and dynamic phase transitions observed across these systems represent distinct asymptotic limits of a singular thermodynamic and kinematic balance.

## 2. Mathematical Formulation

### 2.1. Phenomenological Framework and Kinematics

The foundation of this study is a thermodynamically constrained phenomenological framework for describing the spatiotemporal evolution of macroscopic order in driven, far-from-equilibrium complex fluids. This framework is best understood as a thermodynamically constrained phenomenological synthesis: the specific choice of structural variables, the functional forms of the free energy expansion, and the coupling coefficients are phenomenological in nature, selected based on symmetry considerations, experimental observations, and physical intuition about the systems under investigation. However, these phenomenological choices are embedded within a thermodynamic structure derived from the second law of thermodynamics. The dissipation potential, the flux–force relations, the entropy production inequality, and the fluctuation-dissipation theorem are direct consequences of irreversible thermodynamics. The variational structure of the free energy and the derivation of thermodynamic forces as functional derivatives follow from the principles of statistical thermodynamics. Thus, while the specific material-dependent functions are phenomenological inputs that must be determined or validated for each system, the architectural principles that relate them (objectivity, thermodynamic consistency, non-negative entropy production, and frame invariance) are general inferences from fundamental physical laws. This hybrid approach, combining phenomenological flexibility with thermodynamic, is what enables the framework to describe disparate active matter systems while maintaining predictive capability and physical consistency.

#### 2.1.1. Structural Variables and Kinematic Foundations

We consider a driven, far-from-equilibrium medium whose structural state is characterized by a coarse-grained, symmetric, traceless second-rank tensor $Q\left(r,t\right)$. This tensor describes the orientational (quadrupolar) anisotropy of the constituents, providing a mesoscopic representation of the local structural organization that generalizes the classical nematic director description to accommodate biaxiality and topological defects. The choice of a tensorial order parameter is phenomenological in origin. It is selected because it is the lowest-rank symmetric traceless tensor that captures the essential symmetries of the nematic phase while accommodating biaxiality and topological defects. This choice is validated by the success of the Landau–de Gennes paradigm in describing liquid crystalline phases and is adopted here as the foundational structural descriptor.

To attain sequential symmetry-breaking cascades, we introduce two auxiliary fields that extend the descriptive capacity of the framework beyond tensorial order. The first is a conserved scalar field $\phi \left(r,t\right)$, which acts as a generalized thermodynamic control parameter representing local temperature, concentration, or density, and mediates the primary and secondary instability thresholds that govern the hierarchical emergence of order. The second is an emergent polar vector field $P\left(r,t\right)$, which describes dipolar (vectorial) order that may arise from the tensorial scaffold via a secondary bifurcation, capturing the transition from apolar to polar phases (observed in, for example, ferroelectric liquid crystals and active fluids). The complete thermodynamic state is represented by Q, P and ϕ, evolving under a macroscopic drift velocity $U\left(r,t\right)$ that advects and deforms the structural fields.

Any evolution law for a material tensor must be objective, meaning it must remain invariant under superimposed rigid-body rotations of the observer frame [24]. This requirement is a fundamental constraint on any continuum description, derived from the principle of material frame indifference. The simplest objective derivative that transports and rotates the tensor with the flow is the corotational (Jaumann) rate [24]:

(1) $$\dot{Q}\equiv \frac{\partial Q}{\partial t}+U\cdot \nabla Q-\Omega \cdot Q+Q\cdot \Omega$$

where $\dot{Q}$ represents the corotational rate of the tensor field *Q*, and Ω is the antisymmetric vorticity tensor $\Omega_{ij}=\frac{1}{2}\left(\partial_{j}U_{i}-\partial_{i}U_{j}\right)$ that accounts for the local spin of the continuum. In the most general formulation, the Jaumann rate may be augmented by a flow-alignment term proportional to the symmetric rate-of-strain tensor, as established in the Beris–Edwards thermodynamic framework for complex fluids [10]. But in the strongly driven, active regimes that are the focus of this study, where active torques and thermodynamic restoring forces dominate over shear-induced alignment, we take the flow-tumbling limit in which this additional contribution vanishes. We therefore adopt Equation (1) as the fundamental kinematic flux. The reintroduction of the flow-alignment term is straightforward and does not alter the thermodynamic structure developed below.

The interaction between the structural anisotropy *Q* and an external or internal flux *J* (representing a thermal, chemical, or electrical current) is described by the lowest-order scalar invariant that preserves inversion symmetry J→−J and rotational invariance. This phenomenological coupling takes the form:

(2) $$F_{\mathrm{active}}=-\alpha \int_{V}Q:{\left(J⊗J\right)}^{ST}\;d^{3}r$$

where

(3) $${\left(J⊗J\right)}_{ij}^{ST}=J_{i}J_{j}-\frac{1}{3}|J{|}^{2}\delta_{ij}$$

is the symmetric-traceless projection of the dyadic product, and α is a phenomenological coupling constant that determines the strength and sign of the active alignment. The projection onto the traceless subspace is essential because *Q* is traceless by construction. The isotropic part of the dyadic product would yield a null contraction with *Q*. This coupling is phenomenological in the sense that it is the simplest term consistent with the required symmetries, but it is motivated by the physical observation that external fluxes impose directional anisotropy on the medium.

#### 2.1.2. Thermodynamic Consistency and Dissipation Potential

Having established the kinematic and coupling structure, we now introduce the thermodynamic constraints that ensure the framework is consistent with the second law of thermodynamics. This is the point at which the construction transitions from being phenomenological to being thermodynamically constrained.

The corresponding thermodynamic force conjugate to *Q* is the negative functional derivative:

(4) $$H_{\mathrm{active}}\equiv -\frac{\delta F_{\mathrm{active}}}{\delta Q}=\alpha {\left(J⊗J\right)}^{ST}$$

This active torque acts as a driving force that aligns the principal axes of *Q* with the eigenframe of J⊗J, providing the mechanism through which external fluxes impose structural anisotropy on the medium. For the complete coupled system comprising the tensorial order parameter *Q*, the polar vector field *P*, the conserved scalar field ϕ, and the macroscopic flow velocity *U*, the total local entropy production rate is the sum of contributions from each dissipative mechanism:

(5) $${\dot{s}}_{\mathrm{total}}=H_{\mathrm{total}}:\dot{Q}+H_{P}\cdot \dot{P}+H_{\phi }\dot{\phi }+\frac{1}{T}\sigma :\nabla U+\frac{q\cdot \nabla T}{T^{2}}\geq 0$$

where the terms represent, respectively, the tensorial relaxation contribution, the polar relaxation contribution, the scalar diffusion contribution, the viscous dissipation contribution, and the thermal conduction contribution. The conjugate force for the polar field is $H_{P}=-\delta F_{\mathrm{total}}/\delta P$, the conjugate force for the scalar field is $H_{\phi }=-\delta F_{\mathrm{total}}/\delta \phi$, σ is the total stress tensor (comprising viscous, elastic, and active contributions), *q* is the heat flux, and *T* is the local temperature. In the presence of additional dissipative processes (such as chemical reactions, electric currents, or external flux transport) corresponding terms must be appended to Equation (5).

Thermodynamic consistency of the complete system requires that each flux–force pair be related through a positive-definite mobility matrix. For the tensorial contribution, this is satisfied by the convexity of the dissipation potential $\Psi \left(\dot{Q}\right)$ and the resulting flux–force relation $H_{\mathrm{total}}=\partial \Psi /\partial \dot{Q}$, which guarantees ${\dot{s}}_{Q}=H_{\mathrm{total}}:\dot{Q}\geq 0$. For the polar field, the slaving approximation $\partial f_{\mathrm{bulk}}/\partial P=0$ implies that the polar relaxation is fast and its contribution to entropy production is non-negative provided the polar mobility is positive. For the scalar field, the Cahn–Hilliard equation with positive mobility $M_{\phi }>0$ ensures ${\dot{s}}_{\phi }=H_{\phi }\dot{\phi }\geq 0$ when the Onsager relation $\dot{\phi }=-M_{\phi }\nabla^{2}\left(\delta F/\delta \phi \right)$ is satisfied. For the flow field, viscous dissipation is positive when the viscosity η>0, and active contributions to the entropy production are incorporated through the active stress term, which can be positive or negative depending on the sign of the activity coefficient ζ. The thermal conduction contribution is positive when the thermal conductivity is positive.

In the present formulation, the primary focus is on the tensorial contribution ${\dot{s}}_{Q}=H_{\mathrm{total}}:\dot{Q}\geq 0$ because the structural tensor *Q* is the principal order parameter governing the symmetry-breaking transitions. However, the full thermodynamic consistency of the complete coupled system is verified by ensuring that all mobility coefficients and transport coefficients are positive-definite, that the dissipation potential is convex, and that the complete set of flux–force relations satisfies the Onsager reciprocal relations in the linear regime. The slaving approximation for *P* and the Cahn–Hilliard structure for ϕ are thermodynamically admissible because they correspond to the adiabatic elimination of fast variables while preserving the non-negativity of the entropy production in the reduced description. This hierarchical approach to thermodynamic consistency, focusing on the dominant dissipative contribution while ensuring the auxiliary fields satisfy their own non-negative entropy production conditions, is the foundation of the thermodynamic framework developed in this study.

To assure non-negative entropy production, we introduce a dissipation potential $\Psi \left(\dot{Q}\right)$ that is a convex function of the flux and satisfies $\Psi \left(0\right)=0$, whose derivative with respect to $\dot{Q}$ also vanishes at the origin. In the standard Onsagerian formalism [25], the thermodynamic force is given by:

(6) $$H_{\mathrm{total}}=\frac{\partial \Psi }{\partial \dot{Q}}$$

Equation (6) defines the flux as a function of the force via the Legendre transform of the dissipation potential. This relation is a thermodynamic deduction, given a convex dissipation potential, the flux–force relation that maximizes entropy production (or, equivalently, minimizes the dissipation at fixed force) is obtained by this variational principle.

The identification of $\dot{Q}$ as the thermodynamic flux follows directly from the structure of the entropy production inequality. In the formalism of irreversible thermodynamics, the local entropy production rate is given by the sum over all conjugate flux–force pairs, each representing a distinct dissipative mechanism. Here, $Q\left(r,t\right)$ serves as the structural state variable of the mesoscopic medium. Its rate of change constitutes the kinematic flux naturally conjugated to the thermodynamic force $H_{\mathrm{total}}=-\delta F_{\mathrm{total}}/\delta Q$. This conjugate pairing is validated by the entropy production inequality $\dot{s}=H_{\mathrm{total}}:\dot{Q}\geq 0$, which assures that the product of force and flux yields the local entropy production rate. The corotational (Jaumann) derivative in Equation (1) is specifically chosen over the simple partial time derivative to ensure objectivity. The flux must transform covariantly under superimposed rigid-body rotations of the observer frame, which is essential for a material tensor embedded in a flowing continuum. The Jaumann rate (Equation (1)) represents the rate of change of the structural tensor as measured by an observer rotating with the local fluid vorticity, making it the appropriate kinematic flux for a tensorial order parameter in a macroscopic flow. The dissipation potential $\Psi \left(\dot{Q}\right)$ formalizes this conjugate relationship by establishing the general nonlinear flux–force relation $H_{\mathrm{total}}=\partial \Psi /\partial \dot{Q}$, which is ensured by the convexity of Ψ to produce non-negative entropy production. In the linear limit, this reduces to the familiar closure $\dot{Q}=\Gamma_{0}H_{\mathrm{total}}$, recovering the standard Onsagerian form while preserving the objective tensorial structure of the flux. Thus, the corotational rate $\dot{Q}$ is selected as the thermodynamic flux because it is the objective rate of change of the structural state variable whose conjugate force drives the medium toward thermodynamic equilibrium under the constraints of frame invariance.

If we temporarily specialize to a simple quadratic potential:

(7) $$\Psi \left(\dot{Q}\right)=\frac{1}{2\Gamma_{0}}\dot{Q}:\dot{Q}$$

then Equation (6) yields the linear closure:

(8) $$H_{\mathrm{total}}=\frac{1}{\Gamma_{0}}\dot{Q}⟺\dot{Q}=\Gamma_{0}H_{\mathrm{total}}$$

where $\Gamma_{0}$ is a baseline phenomenological mobility coefficient with dimensions of inverse time per unit energy density. In a more general way, it can be written as:

(9) $$\dot{Q}=\Phi \left(H_{\mathrm{total}}\right)\;$$

where Φ is a monotone operator determined by the choice of Ψ. The linear case corresponds to $\Phi \left(H\right)=\Gamma_{0}H$. By keeping Ψ arbitrary, this framework can describe highly driven systems where the flux saturates or exhibits power-law behavior, such as $\Phi \left(H\right)=\Gamma_{0}|H{|}^{n-1}H$ for power-law fluids or $\Phi \left(H\right)=\Gamma_{0}\left(|H|-H_{y}\right)\;H/|H|$ for Bingham-type yield-stress materials.

For the rest of this study, we depart from the simple linear near-equilibrium approximation and adopt a state-dependent mobility closure to capture the saturation of structural relaxation in densely packed, highly ordered active systems. This closure is phenomenological. It is motivated by the physical observation that in dense systems, the available free volume diminishes as order increases, leading to kinetic arrest. It is constrained by thermodynamics, the closure must satisfy $\dot{s}=H_{\mathrm{total}}:\Phi \left(H_{\mathrm{total}},S\right)\geq 0$, which requires $\Gamma \left(S\right)\geq 0$. We replace the constant mobility with the nonlinear, order-dependent form:

(10) $$\Phi \left(H_{\mathrm{total}},S\right)=\Gamma \left(S\right)\;H_{\mathrm{total}}$$

with $\Gamma \left(S\right)=\Gamma_{0}\left(1-S^{2}/S_{\max}^{2}\right)$ and where $\Gamma_{0}$ is the mobility, $S=\sqrt{\frac{3}{2}\mathrm{Tr}\left(Q^{2}\right)}$ is the local scalar order parameter mapping the magnitude of nematic alignment, and $S_{\max}$ represents the maximum physically attainable alignment. This form encodes the observation that as the system becomes highly ordered ($S\to S_{\max}$), the available free volume diminishes and particles effectively jam, causing a localized kinetic arrest that reduces their ability to structurally relax or align to the applied active torque. This closure keeps the thermodynamic consistency condition $\dot{s}=H_{\mathrm{total}}:\Phi \left(H_{\mathrm{total}},S\right)\geq 0$ provided $\Gamma \left(S\right)\geq 0$, which is satisfied for all physically accessible states with $S\leq S_{\max}$.

The state-dependent jamming closure introduced in Equation (10) necessitates a closer look at the convexity of the dissipation potential. The dissipation potential $\Psi \left(\dot{Q}\right)$ was initially introduced as a convex function of the kinematic flux, with the quadratic case $\Psi \left(\dot{Q}\right)=\frac{1}{2\Gamma_{0}}\dot{Q}:\dot{Q}$ providing a globally convex example. For this quadratic potential, global convexity is guaranteed since the second variation $\delta^{2}\Psi =\frac{1}{\Gamma_{0}}\delta \dot{Q}:\delta \dot{Q}\geq 0$ is positive for $\Gamma_{0}>0$. The flux–force relation derived from this potential yields the linear closure $\dot{Q}=\Gamma_{0}H_{\mathrm{total}}$, which satisfies non-negative entropy production and the Onsager reciprocal relations.

For the state-dependent jamming closure $\Phi \left(H_{\mathrm{total}},S\right)=\Gamma \left(S\right)H_{\mathrm{total}}$ with $\Gamma \left(S\right)=\Gamma_{0}\left(1-S^{2}/S_{\max}^{2}\right)$, the dissipation potential is obtained by the Legendre transform $\Psi \left(\dot{Q},S\right)={sup}_{H}\left[H:\dot{Q}-\frac{1}{2}\Gamma {\left(S\right)}^{-1}H:H\right]$, yielding $\Psi \left(\dot{Q},S\right)=\frac{1}{2}\Gamma {\left(S\right)}^{-1}\dot{Q}:\dot{Q}$ when $\Gamma \left(S\right)>0$. The convexity of this potential with respect to the flux $\dot{Q}$ requires $\delta^{2}\Psi =\Gamma {\left(S\right)}^{-1}\delta \dot{Q}:\delta \dot{Q}\geq 0$, which is satisfied when $\Gamma \left(S\right)>0$. For all physically accessible states with $S<S_{\max}$, we have $\Gamma \left(S\right)>0$, and the potential is locally convex in the flux variable.

It is also important to recognize that global convexity of the dissipation potential in the full mathematical space of all possible fluxes is not guaranteed when the mobility depends on the order parameter *S*, because *S* itself evolves with the flux through the tensorial dynamics. The convexity must be understood in the context of the complete thermodynamic state space, where the dissipation potential is a function of both the flux $\dot{Q}$ and the current structural state *Q* (through the scalar order *S*). The relevant condition for thermodynamic consistency is not global convexity of Ψ in the unrestricted sense, but local convexity along physically realizable trajectories (those that satisfy the evolution equation and the constraints $0\leq S\leq S_{\max}$). Within this physically accessible domain, the jamming function $\Gamma \left(S\right)=\Gamma_{0}\left(1-S^{2}/S_{\max}^{2}\right)$ is positive, and the dissipation potential is convex in the flux variable. This is sufficient to guarantee the monotonicity of the flux–force relation, the non-negativity of entropy production $\dot{s}=H_{\mathrm{total}}:\Phi \left(H_{\mathrm{total}},S\right)=\Gamma \left(S\right)|H_{\mathrm{total}}{|}^{2}\;\geq \;0$, and the satisfaction of the second law of thermodynamics along all admissible paths.

In the limit $S\to S_{\max}$, the mobility $\Gamma \left(S\right)\to 0$, and the dissipation potential becomes singular in the sense that its inverse diverges. This corresponds to the kinetic arrest regime where the structural degrees of freedom are frozen and no further flux is possible. The potential is no longer convex at this singular point, but this is consistent with the interpretation of the jamming transition as a dynamic phase boundary rather than a violation of thermodynamic principles. For all states with $S<S_{\max}$, which includes the entire pre-jamming regime of interest in this study, the dissipation potential remains convex, and the thermodynamic consistency of the framework is maintained. This hierarchical approach to convexity (requiring local convexity on the physically accessible manifold rather than global convexity in the unrestricted mathematical space) is standard in the thermodynamics of complex materials with state-dependent transport coefficients and is consistent with the generalized Onsager formalism for systems with order-parameter-dependent mobilities.

#### 2.1.3. Free Energy Landscape and Coupling Structure

The equilibrium-like part of the thermodynamics is encoded in a total free energy functional that depends on *Q*, *P*, ϕ and their spatial gradients [22]:

(11) $$F_{\mathrm{total}}=\int_{V}\left[f_{\mathrm{bulk}}\left(Q,P,\phi \right)+f_{\mathrm{grad}}\left(\nabla ,Q,\nabla P\nabla \phi \right)\right]d^{3}r$$

The specific functional forms of the free energy contributions represent the phenomenological input to the framework. They are selected based on symmetry considerations, experimental observations of the phase behavior of liquid crystals and active fluids, and the physical requirements of thermodynamic stability. Notice that the framework is modular. These functional forms can be replaced or extended to describe different material systems without altering the underlying thermodynamic-kinematic architecture.

Before presenting the explicit mathematical forms of individual contributions, it is important to articulate how the total free energy functional depends on *Q*, *P* and ϕ. The functional $F_{\mathrm{total}}\left[Q,P,\phi \right]$ represents a hierarchical structure in which each field plays a distinct thermodynamic role. The tensorial contribution depends on the orientational order parameter *Q* through its scalar invariants $\mathrm{Tr}\left(Q^{2}\right)$ and $\mathrm{Tr}\left(Q^{3}\right)$, with coefficients $A_{Q}\left(\phi \right)$, B, C, and E that control the depth and shape of the nematic potential. The polar contribution depends on the vector field *P* through its magnitude $|P{|}^{2}$, $|P{|}^{4}$, and $|P{|}^{6}$, with coefficients $A_{P}\left(\phi \right)$, D, and $E_{P}$. The coupling term depends on both *Q* and *P* through the scalar contraction $Q:{\left(P⊗P\right)}^{ST}$, which measures the alignment between the tensorial anisotropy axis and the dipolar orientation. The coupling coefficient $\gamma \left(S\right)=\gamma_{0}e^{-\lambda S}$ depends on the scalar order $S=\sqrt{\frac{3}{2}\mathrm{Tr}\left(Q^{2}\right)}$ derived from *Q*, introducing an additional implicit dependence through the tensorial field. This exponential decay encodes the physical mechanism by which steric crowding in highly ordered regions attenuates the efficiency of tensor-polar coupling. The gradient contribution depends on the spatial derivatives of all three fields through the square-gradient terms, introducing finite correlation lengths that regularize the formation of spatial patterns and domain structures. It is important to note that the scalar field ϕ acts as the primary thermodynamic control parameter that modulates the tensorial and polar instability thresholds through the linear dependences $A_{Q}\left(\phi \right)=a_{Q}\left(\phi -\phi_{c}^{Q}\right)$ and $A_{P}\left(\phi \right)=a_{P}\left(\phi -\phi_{c}^{P}\right)$, with $\phi_{c}^{Q}>\phi_{c}^{P}$ to enforce a sequential cascade from tensorial to polar order. This hierarchical modulation ensures that as ϕ is varied (by cooling in the liquid crystal system or by increasing the particle area fraction in the colloidal system) the system traverses a sequence of symmetry-breaking transitions which is isotropic → nematic → polar. Thus, free energy landscape encodes the full thermodynamic competition between orientational ordering, dipolar alignment, packing constraints, and spatial gradients, providing the variational foundation from which the thermodynamic forces acting on each field are derived.

The bulk density is taken as a generalized Landau–de Gennes expansion that includes the tensorial contribution:

(12) $$f_{Q}=\frac{A_{Q}\left(\phi \right)}{2}\mathrm{Tr}\left(Q^{2}\right)-\frac{B}{3}\mathrm{Tr}\left(Q^{3}\right)+\frac{C}{4}{\left[\mathrm{Tr}\left(Q^{2}\right)\right]}^{2}+\frac{E}{6}{\left[\mathrm{Tr}\left(Q^{2}\right)\right]}^{3}$$

the polar contribution:

(13) $$f_{P}=\frac{A_{P}\left(\phi \right)}{2}|P{|}^{2}+\frac{D}{4}|P{|}^{4}+\frac{E_{P}}{6}|P{|}^{6}$$

and the bilinear coupling between tensor and vector fields:

(14) $$f_{\mathrm{int}}=-\gamma \left(S\right)\;Q:{\left(P⊗P\right)}^{ST}$$

The gradient terms are taken as the simplest square-gradient [26]:

(15) $$f_{\mathrm{grad}}=\frac{L}{2}\left(\partial_{k}Q_{ij}\right)\left(\partial_{k}Q_{ij}\right)+\frac{K}{2}|\nabla P{|}^{2}+\frac{\kappa }{2}|\nabla \phi {|}^{2}$$

where *L*, *K* and κ are positive stiffness coefficients that penalize sharp spatial variations and introduce finite correlation lengths. The *L* term corresponds to the one-constant elastic approximation for the nematic director. Notice that anisotropic elastic constants can be incorporated by replacing *L* with a tensor of Frank constants without altering the thermodynamic structure.

The total thermodynamic force acting on *Q* is therefore the variational derivative:

(16) $$H_{\mathrm{total}}=-\frac{\delta F_{\mathrm{total}}}{\delta Q}=\alpha {\left(J⊗J\right)}^{ST}-\frac{\partial f_{\mathrm{bulk}}}{\partial Q}+L\nabla^{2}Q$$

where the first term is the active torque (Equation (4)), the second is the bulk restoring force derived from the expanded potentials in Equations (12)–(14), and the third is the elastic diffusion. This equation is independent of the dissipation potential and remains valid in both linear and nonlinear closures.

Thermal or active fluctuations are introduced by adding a Gaussian white noise tensor $\xi \left(r,t\right)$ to the thermodynamic force. The noise is symmetric, traceless, and satisfies the fluctuation-dissipation relation [27,28]:

(17) $$\left\langle \xi_{ij}\left(r,t\right)\xi_{kl}\left(r^{\prime },t^{\prime }\right)\right\rangle =2\Gamma \left(S\right)k_{B}T_{\mathrm{eff}}\left(\delta_{ik}\delta_{jl}+\delta_{il}\delta_{jk}-\frac{2}{3}\delta_{ij}\delta_{kl}\right)\delta \left(r-r^{\prime }\right)\delta \left(t-t^{\prime }\right)$$

where $\Gamma \left(S\right)$ is the state-dependent mobility coefficient, $k_{B}$ is Boltzmann’s constant, and $T_{\mathrm{eff}}$ is an effective temperature that parametrizes the intensity of non-equilibrium fluctuations in the driven medium. In the equilibrium limit, $T_{\mathrm{eff}}\to T$, and Equation (17) reduces to the standard fluctuation-dissipation theorem. For active systems, $T_{\mathrm{eff}}$ may exceed the ambient temperature, reflecting the additional continuous energy injection from microscopic driving forces. For our nonlinear closure, the noise amplitude naturally incorporates the state-dependent jamming prefactor via multiplicative noise to preserve structural fluctuations near kinetic arrest. The fluctuation-dissipation relation is a thermodynamic deduction, not a phenomenological assumption. It follows from the requirement that the system approaches the correct equilibrium distribution in the absence of external driving.

#### 2.1.4. Complete Coupled Evolution Equations

Combining Equations (1), (10), (16) and (17), we obtain the generalized tensorial evolution equation:

(18) $$\tau \dot{Q}=\Phi \left(H_{\mathrm{total}},S\right)+\xi$$

which, under state-dependent dissipative closure, expands to:

(19) $$\tau \dot{Q}=\Gamma \left(S\right)\left[\alpha {\left(J⊗J\right)}^{ST}-\frac{\partial f_{\mathrm{bulk}}}{\partial Q}+L\nabla^{2}Q\right]+\xi$$

where τ is the memory time introduced to give the equation dimensionally homogeneous and to encode the intrinsic relaxation time of the structural field. Although τ and the baseline mobility $\Gamma_{0}$ appear as separate parameters, they are linked in the linear limit where the effective relaxation rate becomes $\Gamma_{0}/\tau$. We retain both quantities to allow independent tuning of the characteristic relaxation time (which may arise, for instance, from rotational viscosity) and the dissipative coupling strength.

Expanding the bulk derivative terms using the modified free energy yields:

(20) $$\tau \dot{Q}=\Gamma \left(S\right)\left[\alpha {\left(J⊗J\right)}^{ST}-A_{Q}\left(\phi \right)Q+B\left(Q^{2}-\frac{1}{3}\mathrm{Tr}\left(Q^{2}\right)I\right)-C\mathrm{Tr}\left(Q^{2}\right)Q-E{\left[\mathrm{Tr}\left(Q^{2}\right)\right]}^{2}Q+\gamma \left(S\right){\left(P⊗P\right)}^{ST}+L\nabla^{2}Q\right]+\xi$$

Equation (20) is the complete dynamical expression. Note that in evaluating the variational derivative of the interaction energy, we treat the order-dependent coupling attenuation $\gamma \left(S\right)$ as a local quasi-static parameter during the thermodynamic relaxation step. This omits the secondary derivative with respect to *Q*, reflecting the assumption that steric crowding modulates the local cross-coupling efficiency without generating an independent, higher-order structural restoring force.

Equation (20) must be supplemented by the dynamical equations for the auxiliary fields to form a closed system suitable for numerical integration. For the polar vector field P we introduce a fully dynamic relaxational evolution equation. To ensure material frame indifference under macroscopic flow, the polar vector evolves via the corotational derivative $\dot{P}=\frac{\partial P}{\partial t}+U\cdot \nabla P-\Omega \cdot P$. The relaxational dynamics follow the standard Onsagerian flux–force relation $\tau_{P}\dot{P}=\Gamma_{P}H_{P}+\xi_{P}$, where $H_{P}=-\delta F_{total}/\delta P$. Expanding the variational derivative yields the local constitutive evolution equation:

(21) τP∂P∂t+U⋅∇P−Ω⋅P=ΓP−APϕP−D|P|P2−EP|P|P2+2γSQ⋅P+K∇2P+ξP

where $\tau_{P}$ and $\Gamma_{P}$ are the intrinsic memory time and mobility of the polar field, respectively, and $K\nabla^{2}P$ provides spatial regularization for polar domains. This dynamic formulation ensures that as the system approaches the critical threshold $\phi_{c}^{P}$ and the thermodynamic restoring force diminishes, the structural relaxation naturally slows down without mathematical singularity.

The conserved scalar field ϕ, representing local density, concentration, or temperature, evolves according to a Cahn–Hilliard-type diffusion equation that accounts for advection, diffusive transport, and coupling to the structural order:

(22a) $$\frac{\partial \phi }{\partial t}+U\cdot \nabla \phi =\nabla \cdot \left(M_{\phi }\nabla \frac{\delta F_{\mathrm{total}}}{\delta \phi }\right)-\nabla \cdot J_{\mathrm{active}}$$

where $M_{\phi }$ is a positive-definite mobility coefficient for the scalar field, and $J_{\mathrm{active}}$ is an active flux that arises from the non-equilibrium driving, proportional to Q⋅∇S or P⋅∇ϕ, representing the tendency of structural gradients to drive uphill diffusion and localized mass accumulation. The variational derivative $\delta F_{\mathrm{total}}/\delta \phi$ includes contributions from the bulk free energy coefficients $\partial A_{Q}/\partial \phi$, $\partial A_{P}/\partial \phi$, and the gradient penalty $\kappa \nabla^{2}\phi$. This equation ensures that ϕ is conserved in the absence of active sources, while the active flux term provides the feedback mechanism through which structural order influences the scalar field distribution, enabling pattern formation and phase separation.

The macroscopic drift velocity *U* is governed by the generalized incompressible Navier–Stokes equations for active fluid:

(22b) $$\rho \left(\frac{\partial U}{\partial t}+(U\cdot \nabla )U\right)=-\nabla p+\eta \nabla^{2}U+\nabla \cdot \sigma_{\mathrm{active}}+\nabla \cdot \sigma_{\mathrm{elastic}}$$

(22c) ∇⋅U=0

where ρ is the mass density of the medium, *p* is the hydrodynamic pressure, and η is the shear viscosity. The active stress tensor $\sigma_{\mathrm{active}}$ is proportional to the tensorial order parameter, taking the form $\sigma_{\mathrm{active}}=\zeta Q$, where ζ is an activity coefficient that may be positive (extensile) or negative (contractile). This term provides the coupling between the structural anisotropy and the macroscopic flow, spatial variations in *Q* generate active stresses that drive fluid motion, and the resulting flow advects and deforms the structural field through the Jaumann derivative. The elastic stress tensor $\sigma_{\mathrm{elastic}}$ arises from the spatial gradients of the order parameters and includes contributions from the Frank elastic energy, $\sigma_{\mathrm{elastic}}\propto L{\left(\nabla Q\right)}^{2}+K{\left(\nabla P\right)}^{2}$. The incompressibility condition ensures volume conservation, appropriate for dense fluid systems.

Thus, the complete coupled system consists of: (i) the tensorial evolution Equation (20) for *Q*; (ii) the dynamic evolution Equation (21) for *P*; (iii) the conserved scalar Equation (22a) for ϕ; and (iv) the active Navier–Stokes Equations (22b) and (22c) for *U*. These equations are solved simultaneously using a staggered-grid finite-difference scheme. At each time step, the algebraic relation for *P* is solved locally using for example a Newton–Raphson iteration, the scalar equation and the momentum equation are advanced using an explicit Adams–Bashforth scheme with adaptive time-stepping to maintain numerical stability, and the tensorial equation is updated using a semi-implicit Crank–Nicolson scheme for the elastic diffusion term to handle the stiffness associated with the $L\nabla^{2}Q$ term. The coupling between the fields is implemented through operator splitting: the scalar field provides the control parameters $A_{Q}\left(\phi \right)$ and $A_{P}\left(\phi \right)$ for the free energy, the polar field provides the active torque ${\left(P⊗P\right)}^{ST}$ that drives the tensorial dynamics, and the flow field *U* provides the advection and vorticity that enter through the Jaumann derivative. This numerical strategy ensures that the feedback loop between structural order, scalar transport, and hydrodynamic flow is captured self-consistently, enabling the framework to describe the full spectrum of spatiotemporal phenomena observed in active complex fluids.

#### 2.1.5. Nonlinear Constitutive Regimes

The framework’s modularity is demonstrated by the fact that the same kinematic and thermodynamic structure can accommodate a range of nonlinear constitutive behaviors. While the present study focuses on the state-dependent jamming closure of Equation (10) to capture the saturation of structural relaxation in densely packed active systems, alternative closures can be substituted to describe different material responses. This modularity is a key feature of the framework. The specific phenomenological closure is selected based on the system under investigation, but the thermodynamic-kinematic architecture remains unchanged.

For instance, a generalized power-law constitutive relation takes the form:

(23) $$\Phi^{*}\left(F^{*}\right)=\Gamma_{0}|F^{*}{|}^{n-1}F^{*}$$

where n > 0 is the characteristic power-law exponent and $\Gamma_{0}$ is a dimensionless prefactor that scales the baseline mobility. The standard linear case is recovered when n = 1 and $\Gamma_{0}=1$. For sub-linear exponents (n < 0), the material exhibits shear-thinning behavior, where the effective structural mobility decreases under higher driving forces. For super-linear exponents (n > 0), the material behaves as a shear-thickening medium, showing enhanced relaxation rates under intense driving.

A second major class of non-equilibrium behaviors is depicted by a generalized Bingham-type yield-stress relation:

(24) $$\Phi^{*}\left(F^{*}\right)=\left\{\begin{array}{cc} 0, & |F^{*}|\leq F_{y}^{*}\\ \Gamma_{0}\left(1-\frac{F_{y}^{*}}{\left|F^{*}\right|}\right)F^{*}, & |F^{*}|>F_{y}^{*} \end{array}\right.$$

where $F_{y}^{*}$ is the dimensionless yield stress, acting as an active threshold parameter. Below this critical force threshold $\left(|F^{*}|\leq F_{y}^{*}\right)$, the material behaves as a rigid structural solid with zero kinematic relaxation $\left(\Phi^{*}=0\right)$. Once the thermodynamic driving force exceeds the threshold $\left(|F^{*}|>F_{y}^{*}\right)$, the internal structure yields and flows.

A third class of nonlinear behavior involves a state-dependent mobility of the form:

(25) $$\Phi^{*}\left(F^{*},S^{*}\right)=\Gamma^{*}\left(S^{*}\right)F^{*}$$

where the dimensionless mobility function $\Gamma^{*}\left(S^{*}\right)$ is modulated by the local scalar order parameter $S^{*}=\sqrt{\frac{3}{2}\mathrm{Tr}\left(Q^{*2}\right)}$. The functional form of $\Gamma^{*}\left(S^{*}\right)$ can be adapted to model specific microscopic coupling mechanisms. For example, one can implement a structural resistance model given by $\Gamma^{*}\left(S^{*}\right)=\frac{1}{1+\lambda S^{*2}}$, where structural alignment progressively dampens relaxation rates. For the dense active systems focused on in this study, we implement the kinetic jamming function introduced in Equation (10), which forces the local mobility to vanish as the system approaches perfect alignment ($S^{*}\to 1$).

Each of these alternative nonlinear frameworks preserves the requirement of irreversible thermodynamics. Non-negative local entropy production is guaranteed in the dimensionless domain:

(26) $${\dot{s}}^{*}=F^{*}:\Phi^{*}\left(F^{*},S^{*}\right)\geq 0$$

provided that the underlying dissipation potential $\Psi^{*}\left({\dot{Q}}^{*}\right)$ remains a convex function of the kinematic flux. This consistency ensures that regardless of whether the system undergoes power-law scaling, plastic yielding, or localized kinetic jamming, the non-equilibrium evolution equations remain thermodynamically admissible.

### 2.2. Dimensional Analysis and Scaling Regimes

To facilitate systematic comparisons across disparate soft matter systems, we perform a non-dimensionalization. We introduce characteristic scales for the physical quantities: the macroscopic observational length l_0_, which may represent the system size or the wavelength of the dominant instability; the reference macroscopic drift speed *U*_0_; the characteristic magnitude of the active flux *J*_0_; the characteristic energy density scale of the bulk Landau–de Gennes potential *A*_0_; the natural scale for the tensorial order parameter *Q*_0_, set to the saturation magnitude at the isotropic-nematic transition; the natural scale for the scalar field $\phi_{0}$, chosen so that $A_{Q}\left(\phi \right)∼O\left(A_{0}\right)$; the natural scale for the polar vector *P*_0_, set to the saturation magnitude; and the characteristic magnitude of the thermodynamic force $F_{0}=A_{0}Q_{0}$.

The elimination of arbitrary scaling is achieved by fixing the characteristic magnitude *Q*_0_ of the tensorial order parameter to the thermodynamic saturation scale derived from the bulk potential Equation (12). For a uniform, stationary system in the nematic phase, minimization gives $Q_{\mathrm{eq}}∼\sqrt{A_{Q}/C}$. Setting $Q_{0}=\sqrt{A_{0}/C}$ gives the rescaled order parameter $Q^{*}=Q/Q_{0}$ of order unity in the ordered phase. Similarly, we set $P_{0}=\sqrt{A_{0}/D}$ for the polar vector magnitude and $\phi_{0}=A_{0}/a_{Q}$ for the scalar field, ensuring that all dimensionless fields are of order unity in their respective ordered states. This fixing eliminates any ambiguity in the scaling of the dimensionless control parameters.

We define the dimensionless variables:

$$r=l_{0}r^{*},\;\;t=\left(\frac{l_{0}}{U_{0}}\right)t^{*},\;\;U=U_{0}U^{*},\;\;J=J_{0}J^{*},\;\;Q=Q_{0}Q^{*},\;\;P=P_{0}P^{*},\;\;\phi =\phi_{0}\phi^{*}$$

The spatial and temporal derivatives transform as $\nabla =\left(1/l_{0}\right)\nabla^{*}$, $\partial /\partial t=\left(U_{0}/l_{0}\right)\partial /\partial t^{*}$, $\Omega =\left(U_{0}/l_{0}\right)\Omega^{*}$, and $\dot{Q}=\left(U_{0}Q_{0}/l_{0}\right){\dot{Q}}^{*}$. Substituting these scales into the dimensional evolution Equation (20), the bulk force scales as $A_{0}Q_{0}$, the elastic diffusion scales as $LQ_{0}/l_{0}^{2},$ and the active torque scales as $\alpha J_{0}^{2}$. Dividing the entire equation by the characteristic structural relaxation rate $\Gamma_{0}A_{0}Q_{0}$ yields:

(27) $$\frac{\tau U_{0}}{l_{0}\Gamma_{0}A_{0}}{\dot{Q}}^{*}=\Phi^{*}\left(\frac{\alpha J_{0}^{2}}{A_{0}Q_{0}}{\left(J^{*}⊗J^{*}\right)}^{ST}-\frac{\delta F_{\mathrm{bulk}}^{*}}{\delta Q^{*}}+\frac{1}{{\left(l_{0}\sqrt{A_{0}/L}\right)}^{2}}\nabla^{*2}Q^{*}\right)+\xi^{*}$$

with the expanded dimensionless bulk free energy density given by:

(28) $$\begin{array}{c} F_{\mathrm{bulk}}^{*}=\frac{\phi^{*}}{2}\mathrm{Tr}\left(Q^{*2}\right)-\frac{b}{3}\mathrm{Tr}\left(Q^{*3}\right)+\frac{1}{4}{\left[\mathrm{Tr}\left(Q^{*2}\right)\right]}^{2}+\frac{e}{6}{\left[\mathrm{Tr}\left(Q^{*2}\right)\right]}^{3}+\frac{A_{P}\left(\phi^{*}\right)}{2}{\left|P^{*}\right|}^{2}+\frac{1}{4}{\left|P^{*}\right|}^{4}+\frac{e_{P}}{6}{\left|P^{*}\right|}^{6}-\\ g_{0}e^{-\lambda S^{*}}Q^{*}:{\left(P^{*}⊗P^{*}\right)}^{ST} \end{array}$$

Here, $b=B/\left(A_{0}Q_{0}\right)$, $e=EQ_{0}^{2}/A_{0}$, $e_{P}=E_{P}P_{0}^{2}/A_{0}$, $A_{P}^{*}\left(\phi^{*}\right)=A_{P}\left(\phi^{*}\right)P_{0}^{2}/\left(A_{0}Q_{0}^{2}\right)$, $d=DP_{0}^{4}/\left(A_{0}Q_{0}^{2}\right)$, $g_{0}=\left(\gamma_{0}P_{0}^{2}\right)/\left(A_{0}Q_{0}\right)$, and λ is the dimensionless decay constant tracking the alignment attenuation. The dimensionless force is expressed in units of $A_{0}Q_{0}$, and the self-consistent, state-dependent dimensionless flux–force function is defined as:

(29) $$\Phi^{*}\left(F^{*},S^{*}\right)=\frac{1}{\Gamma_{0}A_{0}Q_{0}}\Phi \left(A_{0}Q_{0}F^{*},S\right)=\left(1-S^{*2}\right)F^{*}$$

This definition ensures that near the structural transition threshold ($S^{*}\to 0$), the operator reduces to the standard linear form $\Phi^{*}\left(F^{*}\right)=F^{*}$, where the structural mobility cancels out completely.

To render the left-hand side dimensionally homogeneous with the right-hand side, we multiply Equation (27) by $\frac{l_{0}\Gamma_{0}A_{0}}{\tau U_{0}}$, obtaining the final dimensionless governing relation:

(30) $${\dot{Q}}^{*}=\frac{1}{\mathrm{De}}\Phi^{*}\left(\epsilon {\left(J^{*}⊗J^{*}\right)}^{ST}-\frac{\delta F_{\mathrm{bulk}}^{*}}{\delta Q^{*}}+\frac{1}{\sigma^{2}}\nabla^{*2}Q^{*}\right)+\frac{1}{\mathrm{De}}\xi^{*}$$

where De and ϵ are the generalized Deborah and active torque numbers, respectively, and σ is the confinement (Ginzburg) parameter.

The dimensionless noise correlation mapping directly from the dimensional Equation (17) becomes:

(31) $$\left\langle \xi_{ij}^{*}\left(r^{*},t^{*}\right)\xi_{kl}^{*}\left(r\prime^{*},t\prime^{*}\right)\right\rangle =D_{\mathrm{eff}}\left(\delta_{ik}\delta_{jl}+\delta_{il}\delta_{jk}-\frac{2}{3}\delta_{ij}\delta_{kl}\right)\delta^{*}\left(r^{*}-r\prime^{*}\right)\delta^{*}\left(t^{*}-t\prime^{*}\right)\;$$

with the effective noise intensity defined as:

(32) $$D_{\mathrm{eff}}=2\left(\frac{k_{B}T_{\mathrm{eff}}}{A_{0}Q_{0}^{2}l_{0}^{3}}\right)\left(\frac{U_{0}}{\Gamma_{0}A_{0}l_{0}}\right)$$

This ratio directly balances the effective non-equilibrium fluctuation energy against the core bulk energy of the ordered phase within an observational volume, scaled by the ratio of advective to baseline relaxation rates. Notice that the structural Deborah number $\left(De=\frac{\tau U_{0}}{l_{0}\Gamma_{0}A_{0}}\right)$ quantifies the ratio of the intrinsic structural memory time to the effective dynamic relaxation timescale of the macroscopic flow. When De≪1, the material relaxes quickly compared to the flow timescale, inducing the structural configuration to track the local thermodynamic state. On the other hand, when De≫1, memory effects dominate, and structural fields are advected downstream without sufficient time to undergo localized relaxation. Because our generalized formulation replaces standard linear theory with a state-dependent mobility closure, this base structural Deborah number is dynamically modulated by the slope of the operator $\Phi^{*}$ as the local medium approaches structural arrest. Besides, the active torque number ($\epsilon =\alpha J_{0}^{2}/\left(A_{0}Q_{0}\right)$) scales the magnitude of the non-equilibrium driving force against the characteristic bulk thermodynamic restoring stress. The numerator $\alpha J_{0}^{2}$ holds dimensions of energy density, while the denominator $A_{0}Q_{0}$ represents the representative structural stress. This number serves as the primary control parameter for the structural transition from a passive, isotropic state to a self-organized flocking phase. When ϵ≪1, the system remains in a passive regime dominated by standard thermodynamics, but when ϵ≥1, active driving overpowers the bulk restoring mechanisms, inducing spontaneous macroscopic ordering and spatial pattern formation.

The confinement parameter ($\sigma =l_{0}\sqrt{A_{0}/L}$) compares the observational system scale $l_{0}$ to the intrinsic nematic correlation length $\xi =\sqrt{L/A_{0}}$. When σ≫1 ($l_{0}\gg \xi$), the system is compared to the interfacial boundaries, gradient penalties are minimal, and multiple structural domains are free to form. When σ∼1, spatial confinement is deep, and the global morphology is restricted by topological defects and boundaries. When σ≪1, elastic energy costs are high that any coherent structural alignment is entirely suppressed. Notice that the baseline mobility parameter does not enter this static length scale. It governs only the dynamic response rates. In addition, the cubic coefficient ($b=B/\left(A_{0}Q_{0}\right)$) states the strength of the asymmetric term in the Landau–de Gennes potential, shifting the transition from continuous (second-order) when b=0 to first-order when b>0, and setting the overall depth of the phase coexistence region. The higher-order parameters e and $e_{P}$ impose high thermodynamic penalties that mimic solid volume exclusion as alignment limits are approached.

The baseline tensor-polar coupling ($g_{0}=\gamma_{0}P_{0}^{2}/\left(A_{0}Q_{0}\right)$) controls the initial thermodynamic alignment efficiency between the underlying structural framework and the emergent polar vector field, acting as the dominant mechanism driving the sequential symmetry-breaking cascade. Finally, the effective noise intensity $D_{\mathrm{eff}}$ (Equation (32)) balances fluctuation energy against local volume constraints. When $D_{\mathrm{eff}}\ll 1$, fluctuations are minimal, and the medium follows deterministic trajectory laws. When $D_{\mathrm{eff}}\geq 1$, continuous active energy injection allows non-equilibrium fluctuations to induce nucleation and trigger sudden symmetry-breaking events across the system. The alternative nonlinear closures introduced in Section 2.1.5 preserve their mathematical structure upon non-dimensionalization. For the remainder of this study, we restrict our analysis to the dimensionless state-dependent jamming closure to specifically isolate and quantify the saturation of structural relaxation during densely packed flocking transitions.

### 2.3. Critical Thresholds

A central feature of this framework is that a structural bifurcation is triggered when the active torque number exceeds a critical value of order unity, $\epsilon >\epsilon_{c}≃1$. We now present the derivation of this criterion adapted for our formulation. Consider the homogeneous steady state of the dimensionless system Equation (30) under quiescent conditions where spatial variations, macroflows, and polar fields vanish ($\partial_{t}=0$, ∇=0, U=0, and $P^{*}=0$). Near the isotropic-nematic transition threshold where the order parameter magnitude is small, higher-order steric expansion terms ($e,e_{P}$) are sub-dominant, and Equation (30) reduces to the leading-order balance:

(33) $$0=\Phi^{*}\left(\epsilon T-\phi_{0}^{*}Q_{0}^{*}\right)$$

where $\phi_{0}^{*}$ is the homogeneous scalar control field and $T={\left(J^{*}⊗J^{*}\right)}^{ST}$ represents the symmetric-traceless active flux tensor. Since $\Phi^{*}$ is invertible owing to the monotonicity and convexity of the underlying dissipation potential, the internal thermodynamic force must vanish at steady state, yielding $\epsilon T-\phi_{0}^{*}Q_{0}^{*}=0$. Consequently, the uniform steady-state configuration is given by:

(34) $$Q_{0}^{*}=\frac{\epsilon }{\phi_{0}^{*}}T$$

This homogeneous tracking solution exists for all active torque numbers ϵ>0. To evaluate the stability of this state, consider that the system is perturbed by a small, spatially inhomogeneous fluctuation such that $Q^{*}=Q_{0}^{*}+\delta Qe^{ik\cdot r^{*}+\lambda t^{*}}$ and $\phi^{*}=\phi_{0}^{*}+\delta \phi e^{ik\cdot r^{*}+\lambda t^{*}}$, where *k* is the wavevector and λ is the linear growth rate. Linearizing the generalized evolution Equation (30) about the uniform state, the variation in the bulk term $-\phi^{*}Q^{*}$ with respect to the shifting scalar field yields $-\delta \phi Q_{0}^{*}$. Substituting the steady-state value from Equation (34), this term becomes $-\frac{\epsilon }{\phi_{0}^{*}}T\delta \phi$. Incorporating the Fourier Laplacian $\nabla^{*2}\to -k^{2}$, and recalling the dimensionless structural memory scaling De established in Equation (30), the linearized dynamical equation for the fluctuation reads:

(35) $$De\lambda \delta Q=\Phi_{0}^{\prime }\left(-\phi_{0}^{*}\delta Q-\frac{\epsilon }{\phi_{0}^{*}}T\delta \phi -\frac{k^{2}}{\sigma^{2}}\delta Q\right)$$

where $\Phi_{0}\prime ={\left.\frac{\partial \Phi^{*}}{\partial F^{*}}\right|}_{F_{0}^{*}=0}$ is the generalized mobility tensor evaluated at the zero-force steady state. Notice that, under the state-dependent jamming closure established in Equation (29), this derivative evaluates to $\Phi_{0}\prime =\left(1-S_{0}^{*2}\right)I$, where $S_{0}^{*}$ is the scalar order of the uniform background. For a highly disordered or near-critical baseline system ($S_{0}^{*}\to 0$), we recover $\Phi_{0}\prime \to I$, reducing back to the standard linear closure. In the general active regime, $\Phi_{0}\prime$ remains a symmetric, positive-definite scaling tensor that acts to slow down the relaxation rate as the system approaches structural arrest.

Using the localized thermodynamic relation $\delta \phi \approx \chi Q_{0}^{*}:\delta Q=\chi \frac{\epsilon }{\phi_{0}^{*}}T:\delta Q$, where χ is a feedback coefficient determined by the free energy landscape, the linearized growth rate projected along the principal alignment direction of T (assuming the normalized flux satisfies T:T≈1), the linearized growth rate simplifies to:

(36) $$De\lambda =-\Phi_{0}^{\prime }\left[\phi_{0}^{*}+\frac{k^{2}}{\sigma^{2}}+\chi {\left(\frac{\epsilon }{\phi_{0}^{*}}\right)}^{2}\right]$$

Because the jamming prefactor is positive prior to absolute kinetic arrest ($\Phi_{0}\prime >0$), the linear stability of the uniform state is governed by the sign of the terms within the brackets. When χ>0, the growth rate λ remains negative across all wavenumbers, indicating structural stability. A static structural instability can only arise when the feedback from the scalar field becomes negative (χ<0), representing a destabilising coupling where local scalar depletion occurs in highly ordered zones. This negative cross-coupling is provided by our order-dependent tensor-polar interface $\gamma \left(S\right)$ below the secondary polar threshold.

The full dispersion relation for the coupled Q–ϕ framework yields a classical Turing-type instability. The marginal stability curve separating stable and unstable growth modes (λ=0) requires the bracketed terms of Equation (36) to balance:

(37) $$\phi_{0}^{*}+\frac{k^{2}}{\sigma^{2}}+\chi {\left(\frac{\epsilon }{\phi_{0}^{*}}\right)}^{2}=0$$

For a standard Turing instability to occur at the exact boundary of stability, the feedback must reach a critical negative magnitude of $\chi_{c}=-{\left(\frac{\phi_{0}^{*}}{\epsilon }\right)}^{2}$. Substituting this baseline value into the condition Equation (37) yields:

(38) $$\phi_{0}^{*}+\frac{k^{2}}{\sigma^{2}}-\phi_{0}^{*}=\frac{k^{2}}{\sigma^{2}}=0$$

Equation (43) implies a critical wavenumber of k=0, which corresponds to a uniform global mode rather than a localized finite-wavelength patterning event. For a finite-wavelength Turing pattern to emerge, the destabilizing feedback must overcome this uniform cutoff by an infinitesimal margin. Writing this supercritical feedback as:

(39) $$\chi =-{\left(\frac{\phi_{0}^{*}}{\epsilon }\right)}^{2}\left(1+\eta \right)$$

where η>0 represents the fractional excess of destabilizing feedback beyond the uniform threshold, Equation (37) yields:

(40) $$\phi_{0}^{*}+\frac{k^{2}}{\sigma^{2}}-\phi_{0}^{*}\left(1+\eta \right)=\frac{k^{2}}{\sigma^{2}}-\eta \phi_{0}^{*}=0$$

Solving Equation (40) yields the selection rule for the finite-wavelength instability mode:

(41) $$k^{2}=\eta \phi_{0}^{*}\sigma^{2}$$

Thus, the structural medium spontaneously self-organizes at a finite wavenumber proportional to $\sqrt{\eta }\sigma$. This spatial selection remains independent of the active torque number ϵ, as expected for a static Turing bifurcation where structural geometry is driven by scalar cross-coupling feedback. In contrast, the threshold for a true dynamical bifurcation (where the quiescent uniform state becomes unstable to convective hydrodynamic flows and macro-scale turbulence) requires coupling back to the fluid momentum equations. In active fluids, the internal active torque generates an active stress tensor that drives macroscopic flow fields *U*. The characteristic active Reynolds number ($Re_{active}$) for these self-generated flows scales directly with the active driving force, $Re_{active}\propto \epsilon$. Spontaneous hydrodynamic flow patterns break out when these destabilizing active stresses overwhelm localized viscous dissipation, occurring when $Re_{active}∼O\left(1\right)$. By tracking the higher-order corrections introduced by the zero-force mobility slope, the threshold for dynamical bifurcation follows the generalized criterion:

(42) $$\epsilon >\epsilon_{c}≃\frac{1}{\Phi_{0}^{\prime }}$$

Under our formulation, the baseline critical threshold is shifted by the zero-force mobility slope according to $\epsilon_{c}=1/\Phi_{0}\prime$. Because the jamming function satisfies $\Phi_{0}\prime =1-S_{0}^{*2}∼O\left(1\right)$ for systems initiating from a weakly ordered or isotropic state, the universal threshold condition $\epsilon_{c}≃1$ remains valid as a leading-order approximation across both linear and nonlinear regimes.

### 2.4. Key Elements of the General Framework

Having developed the complete formulation, it is important to articulate the elements that constitute the general framework and distinguish it from prior approaches. These elements, while interconnected, can be understood as a hierarchical sequence of structural decisions that enable the description of sequential symmetry-breaking cascades across disparate active matter systems.

The first key element is the selection of the structural descriptor. The framework employs a single symmetric, traceless second-rank tensor $Q\left(r,t\right)$ as the primary mesoscopic state variable. This tensorial representation generalizes the classical nematic director description by capturing both uniaxial and biaxial order within a unified mathematical object, while incorporating topological defects through the tensor’s spatial variations. Unlike approaches that track separate director vectors or require multiple order parameters for different symmetry classes, the tensorial formulation provides a self-consistent description of orientational anisotropy that is independent of the specific microscopic constituents.

The second key element is the introduction of auxiliary fields that enable sequential symmetry breaking. To extend the descriptive capacity beyond tensorial order, the framework incorporates a scalar field $\phi \left(r,t\right)$ that acts as a generalized thermodynamic control parameter, modulating the instability thresholds through linear coefficients $A_{Q}\left(\phi \right)=a_{Q}\left(\phi -\phi_{c}^{Q}\right)$ and $A_{P}\left(\phi \right)=a_{P}\left(\phi -\phi_{c}^{P}\right)$. This field, representing local temperature, concentration, or packing density, determines the hierarchical emergence of order by ensuring $\phi_{c}^{Q}>\phi_{c}^{P}$, so that tensorial order precedes polar order. A polar vector field $P\left(r,t\right)$ captures dipolar order arising from the tensorial scaffold via a secondary bifurcation, enabling the description of ferroelectric phases and active flocking within the same continuum framework.

The third key element is the objective kinematic framework. The evolution law for any material tensor must be frame-invariant under superimposed rigid-body rotations. The framework adopts the corotational (Jaumann) derivative (Equation (1)) as the kinematic flux, ensuring that the structural dynamics remain objective under macroscopic flow. While the Jaumann rate may be augmented by flow-alignment terms in the general Beris–Edwards formulation, the present framework operates in the flow-tumbling limit appropriate for driven active systems where active torques dominate over shear-induced alignment.

The fourth key element is the thermodynamic foundation through irreversible thermodynamics. The framework is grounded in a convex dissipation potential $\Psi \left(\dot{Q}\right)$ that establishes the general nonlinear flux–force relation (Equation (6)), guaranteeing non-negative entropy production. This thermodynamic consistency is independent of the specific functional form of the flux–force relation, capturing both linear Onsagerian closures and arbitrary nonlinear constitutive behavior. The conjugate thermodynamic force is derived from a total free energy functional that combines bulk, gradient, and coupling contributions, ensuring that the relaxational dynamics respect the underlying free energy landscape.

The fifth key element is the nonlinear state-dependent mobility closure. Departing from standard linear near-equilibrium approximations, the framework introduces a mobility function that depends on the local scalar order parameter $S=\sqrt{\frac{3}{2}\mathrm{Tr}\left(Q^{2}\right)}$. This closure encodes the physical observation that as the system becomes highly ordered, the available free volume diminishes and constituents effectively jam, causing localized kinetic arrest that reduces their ability to structurally relax or align to applied active torques. The mobility vanishes as $S\to S_{\max}$, bounding the order parameters and preventing divergences under extreme active driving.

The sixth key element is the generalized free energy expansion. While the specific polynomial forms in Equations (12)–(14) represent a controlled Taylor expansion in the order parameters about the isotropic reference state, the framework is independent of the detailed functional forms. The free energy includes sixth-order terms with positive coefficients E>0 and $E_{P}>0$ that provide steep energetic penalties at high order parameters, capturing excluded-volume and steric constraints in dense systems.

The seventh key element is the complete evolution equation and its dimensionless parameterization. The generalized tensorial evolution Equation (19) synthesizes active driving, thermodynamic restoring forces, elastic penalties, and stochastic fluctuations into a self-consistent dynamical description. Through systematic non-dimensionalization, the entire far-from-equilibrium state space is compressed into a minimal set of independent dimensionless groups, the active torque number, the Deborah number, the confinement parameter, and the steric and coupling groups. This unified parameterization enables quantitative cross-comparisons between physically disparate systems and reveals the universal scaling laws governing non-equilibrium symmetry breaking.

The eighth key element is the emergence of the active torque number as a universal bifurcation parameter. Linear stability analysis of the homogeneous steady state reveals that structural bifurcations are triggered when the active torque number exceeds a critical value of order unity (Equation (42)). For systems initiating from weakly ordered or isotropic states, the zero-force mobility slope satisfies $\Phi_{0}^{\prime }=1-S_{0}^{*2}∼O\left(1\right)$, yielding the universal threshold condition $\epsilon_{c}≃1$. This criterion governs both static Turing-type instabilities arising from scalar cross-coupling feedback and true dynamical bifurcations driven by active Reynolds numbers, indicating that the transition from isotropic disorder to macroscopic ordered patterns is determined by a thermodynamic-kinematic balance operating independently of specific microscopic interactions.

The ninth key element is the thermodynamic-kinematic synthesis that achieves cross-system generality. The framework synthesizes principles from non-equilibrium irreversible thermodynamics (the dissipation potential and entropy production inequality), generalized Landau–de Gennes expansions (the tensorial order parameter and free energy functional), and active hydrodynamics (the objective kinematic flux and active torque coupling). This synthesis bridges the divide between static equilibrium-based energy minimization and the intrinsically driven mechanics of active systems, establishing a unified methodology for analyzing non-equilibrium soft matter across disparate regimes.

In summary, our framework is distinguished by its tensorial structural description, its hierarchical auxiliary fields enabling sequential symmetry breaking, its objective kinematic formulation, its thermodynamic consistency through convex dissipation potentials, its nonlinear state-dependent mobility closure, its modular free energy architecture, its dimensionless parameterization, and its identification of the active torque number as a universal bifurcation parameter. These elements, operating together, provide a unified phenomenological description of spontaneous macroscopic ordering in driven, far-from-equilibrium complex fluids that transcends the system-specific limitations of prior approaches.

## 3. Spatiotemporal Evolution in Driven Media

### 3.1. General Framework and Experimental Studies

Before presenting the mathematical implementation and results, it is essential to establish how Equation (30) and its associated free energy expansion (Equation (26)) are mapped onto the two distinct experimental systems investigated in this study. This mapping provides the bridge that connects the abstract thermodynamic-kinematic framework of Section 2 to predictions. For the polar liquid crystal system investigated by Gibb et al. [18], the dimensionless scalar field $\phi^{*}$ is identified with the reduced temperature, normalized such that $\phi^{*}=1$ at the isotropic-nematic transition temperature $T_{IN}={154.3\;}^{\circ }C$ and decreasing monotonically with cooling. The primary tensorial instability threshold is set at $\phi_{c}^{Q^{*}}=0.8$, corresponding to the experimentally observed nematic transition, while the secondary polar threshold is set at $\phi_{c}^{P^{*}}=0.5$, corresponding to the onset of ferroelectric order at 111 °C. The active torque number for this system is associated with the internal electric polarization field that arises from the molecular dipoles, with its magnitude controlled by the coupling coefficient α in the active torque term. The confinement parameter $\sigma =l_{0}\sqrt{A_{0}/L}$ is fixed to σ≃50 based on the ratio of the helical pitch (hundreds of nanometers) to the smectic layer spacing (~3 nm). The tensor-polar coupling parameter is set to $g_{0}≃1$, and the exponential decay constant is λ≃2, consistent with the rapid attenuation of polar-nematic coupling in densely packed smectic layers. The cubic coefficient *b* is chosen to reproduce the first-order nature of the isotropic-nematic transition, while the sixth-order coefficients *e* and *e_P_* are set to values that bound the order parameters at saturation. The Deborah number is taken in the limit De≪1, appropriate for the slow cooling rates employed in the experimental protocol, ensuring that the structural fields track the local thermodynamic state. Under these parameter assignments, Equation (30) reduces to a time-dependent Ginzburg–Landau equation for the tensorial order parameter, coupled to the polar vector field through the bilinear interaction term.

For the metal-dielectric Janus colloid system investigated by Das et al. [19], the dimensionless scalar field $\phi^{*}$ is identified with the particle area fraction, normalized by the critical area fraction for the isotropic-hexatic transition. The active torque number is directly proportional to the square of the self-propulsion speed ($\epsilon \propto v_{0}^{2}$) as established by the identification $J\equiv v_{0}\hat{n}$ in the active torque term. The confinement parameter σ is fixed to a value that captures the ratio of the system size to the particle interaction length, while the Deborah number is of order unity to account for the finite relaxation time of the colloidal structure relative to the self-propulsion dynamics. The free energy parameters are chosen to reproduce the purely repulsive nature of the particle interactions, with the sixth-order coefficients *e* and *e_P_* playing a crucial role in capturing the steric constraints that give rise to the hexatic and crystalline phases at high area fractions. The tensor-polar coupling *g*_0_ controls the efficiency with which the tensorial nematic order induces polar alignment, while the exponential decay λ captures the attenuation of this coupling in densely packed regions where turn-away torques dominate.

**Figure 1:**
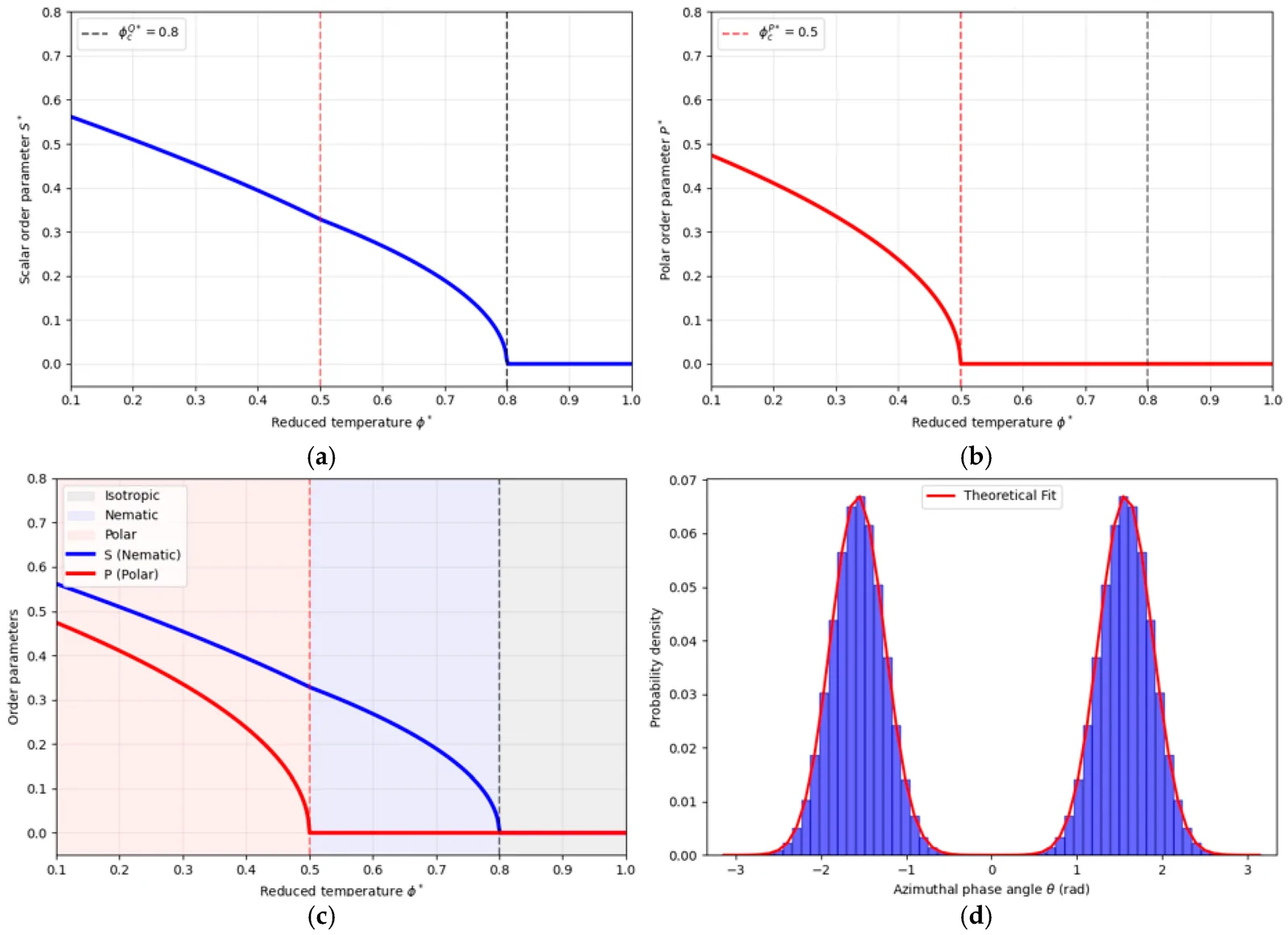
Sequential symmetry-breaking cascade in polar liquid crystals. Panels (**a**–**c**) show the temperature dependence of the scalar nematic order parameter S and polar order parameter P as functions of the reduced temperature $\phi^{*}$. The nematic order occurs at $\phi_{c}^{Q}=0.8$ via a first-order transition, while the polar order emerges at $\phi_{c}^{P}=0.5$ via a second-order transition with $P\propto \sqrt{\phi_{c}^{P}-\phi^{*}}$. Panel (**c**) delineates the isotropic, nematic/SmA, and polar ${\mathrm{SmA}}_{F}$ phase regions. Panel (**d**) shows the azimuthal phase angle histogram for the ${\mathrm{SmC}}_{P}^{H}$ phase, exhibiting two symmetric peaks at 0 and π with equal populations, characteristic of spontaneous $Z_{2}$ symmetry breaking.

**Figure 2:**
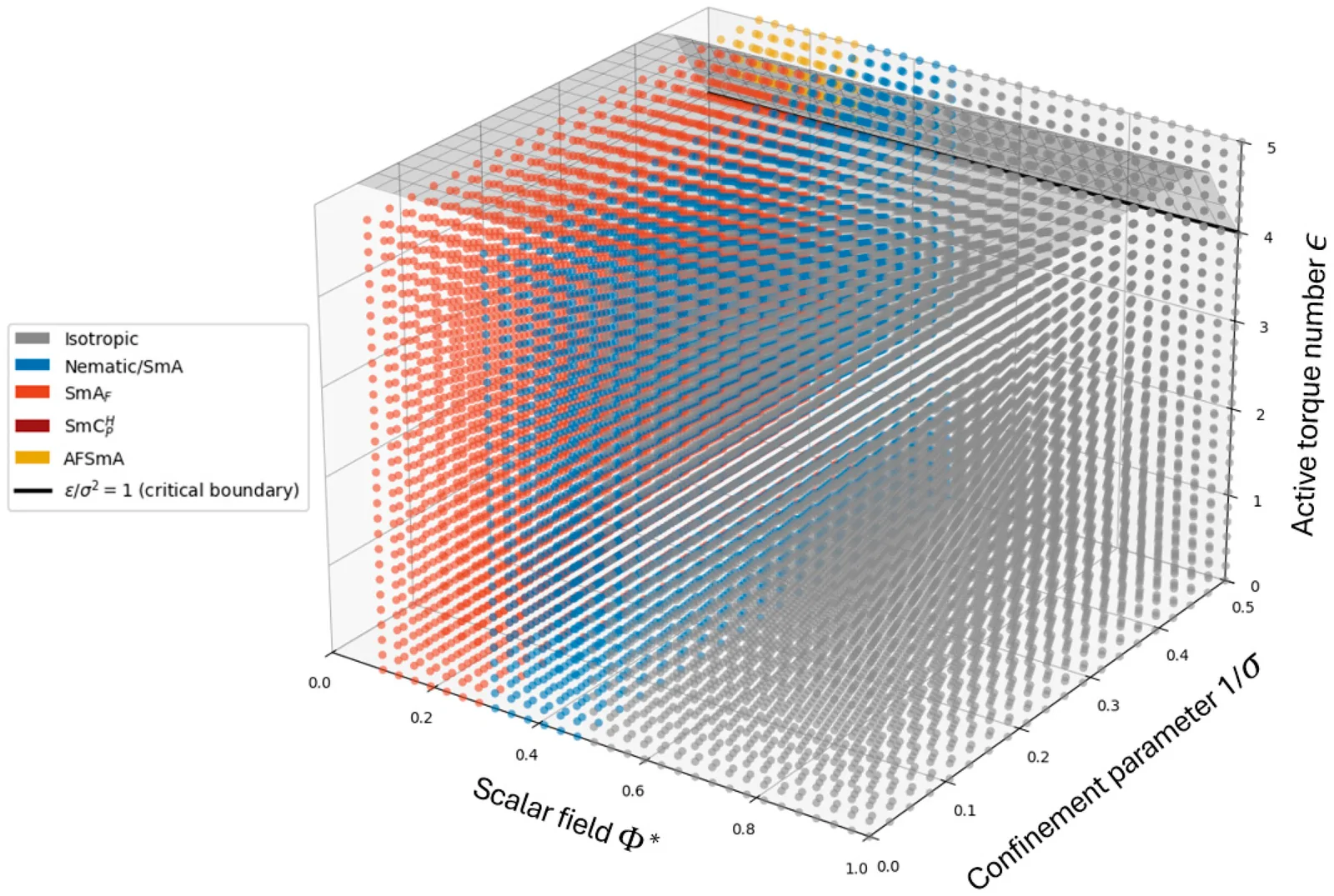
Three-dimensional phase diagram for polar liquid crystals in the dimensionless space. The curved surface at $\epsilon /\sigma^{2}=1$ separates uniform (left) from modulated (right) phases. For $\epsilon <\sigma^{2}$ the system remains uniform and for $\epsilon >\sigma^{2}$ it develops spatial modulations to reduce electrostatic energy. The boundary is drawn with thick black line for clarity.

**Figure 3:**
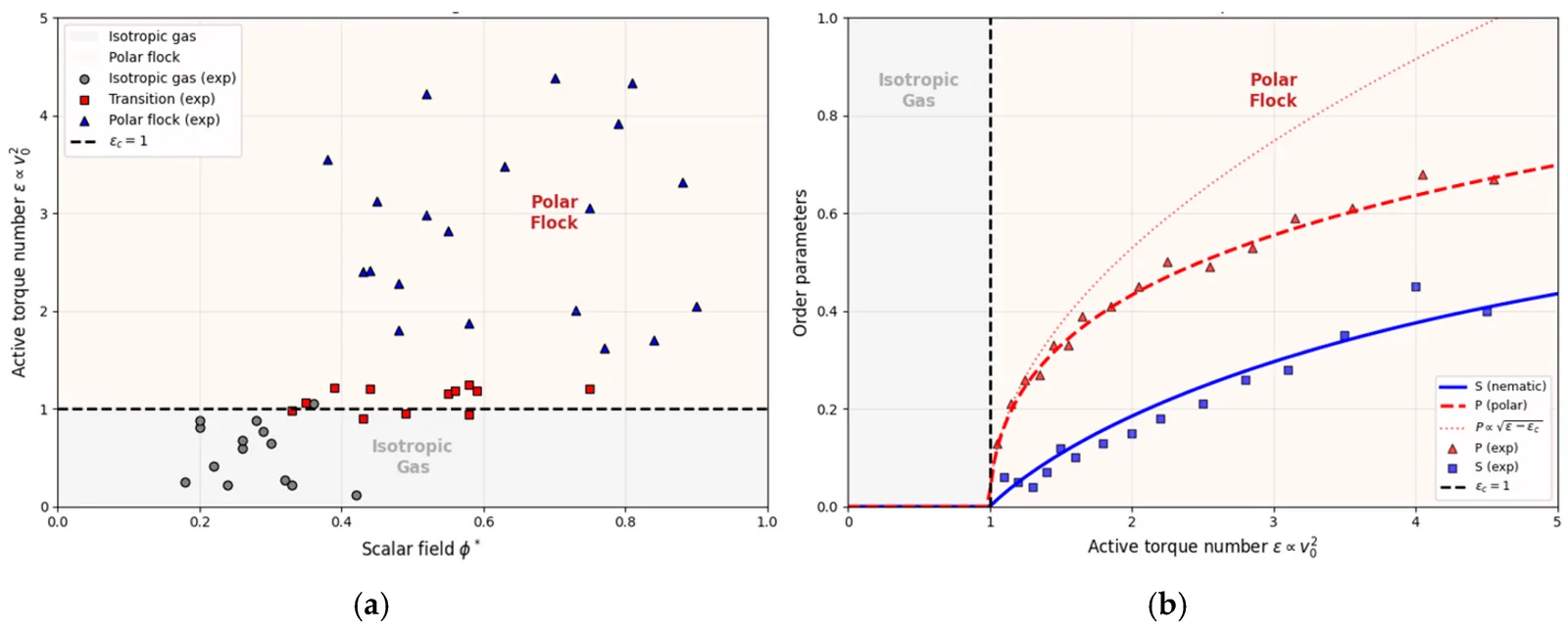
Flocking transition and order parameter dynamics in metal-dielectric Janus colloids: (**a**) Phase diagram mapped onto the two-dimensional dimensionless parameter space of the scalar field $\phi^{*}$ (representing particle area fraction) and the active torque number $\epsilon \propto v_{0}^{2}$. The critical threshold $\epsilon_{c}=1$ (black dashed line) separates the disordered isotropic active gas phase from the macroscopically ordered polar flocking phase; (**b**) Scalar and polar order parameters as a function of the active torque number ϵ. The evolution of the nematic/scalar order parameter S (blue solid line) and the collective polar order parameter P (red dashed line) demonstrates that both structure metrics remain zero in the subcritical active gas regime (ϵ<1). Upon crossing the critical threshold, the system undergoes a continuous structural bifurcation. The pink dotted line illustrates the predicted mean-field Landau expansion scaling law, confirming the second-order nature of the flocking transition with a critical exponent of β=1/2, followed by a smooth saturation of order at high active driving intensities.

**Figure 4:**
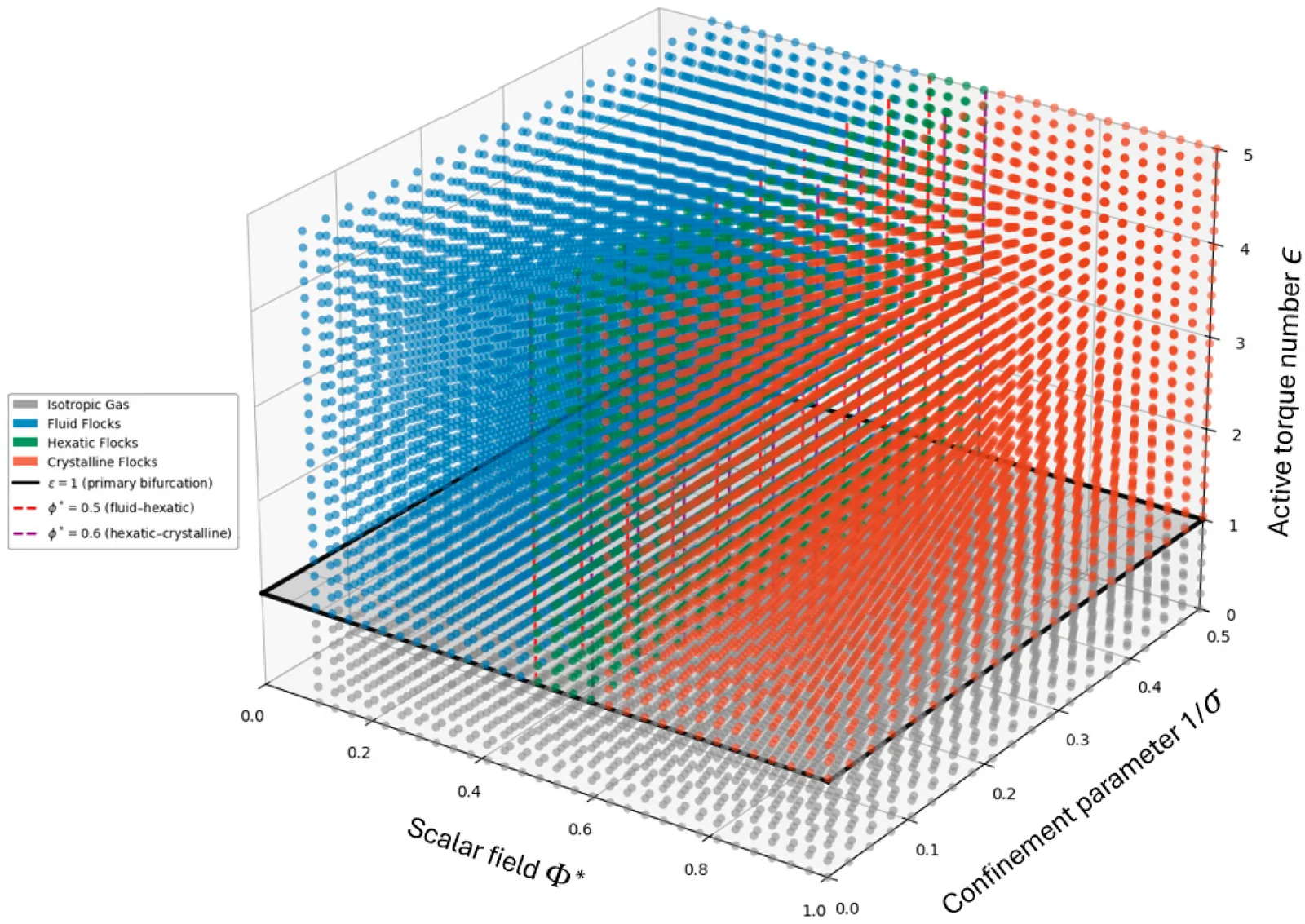
Three-dimensional phase diagram for Janus colloids in the dimensionless parameter space. The black surface at $\epsilon_{c}=1$ marks the primary bifurcation from isotropic to polar order. The red dashed surface at $\phi^{*}=0.5$ separates fluid from hexatic flocks, and the purple dashed surface at $\phi^{*}=0.6$ separates hexatic from crystalline flocks, consistent with the experiments of Das et al. [19], as shown in their Figure 3a.

The correspondence between the predictions and the experimental data is established through the following quantitative relationships. The scalar nematic order parameter $S^{*}$ shown in Figure 1a–c and Figure 3b is computed directly from the tensor contraction $S^{*}=\sqrt{\frac{3}{2}\mathrm{Tr}\left(Q^{*2}\right)}$, with the values obtained from calculations of Equation (30). The polar order parameter $P^{*}$ in Figure 1b–c and Figure 3b is computed from the spatially averaged polar vector field $P^{*}=\left\langle \left|P^{*}\right|\right\rangle$. The phase boundaries in Figure 1c and Figure 3a are determined by locating the values of $\phi^{*}$ and ϵ at which the order parameters cross the threshold $S^{*}=0.05$ and $P^{*}=0.05$, respectively. The azimuthal phase angle histogram in Figure 1d is constructed from the local tilt angle $\psi =arctan\left(P_{y}^{*}/P_{x}^{*}\right)$ extracted from the polar vector field in the ${\mathrm{SmC}}_{p}^{H}$ phase. The three-dimensional phase diagram in Figure 2 is generated by computing the uniform-modulated phase boundary from the condition $\epsilon =\sigma^{2}$, derived from the linear stability analysis of Equation (30). The helical pitch in Figure 2 is computed from the analytical expression $\lambda_{\mathrm{pitch}}∼2\pi \sigma /\sqrt{g_{0}\epsilon -1}$, which follows directly from the wavevector selection rule obtained from the linear stability analysis of the uniform polar state. The block periodicity in the antiferroelectric phase is computed from $p∼2\pi \sigma /\sqrt{\epsilon -\epsilon_{c}}$, derived analogously. The Landau scaling curve in Figure 3b is obtained from $P=\sqrt{\left(\rho \xi \left(\epsilon -\epsilon_{c}\right)-D_{r}\right)/b_{3}}$, which emerges from the mean-field reduction in Equation (30) near the critical threshold. The phase boundaries for the hexatic and crystalline flocks in Figure 4 are determined by the values of $\phi^{*}$ at which the scalar order parameter crosses the thresholds $S^{*}=0.4$ and $S^{*}=0.7$, respectively, corresponding to the experimental identification of these phases in the Janus colloid system.

The parameter values employed for each system are summarized in Table 1, which provides a direct reference for the correspondence between the theoretical parameters and the experimental observables.

### 3.2. Numerical Methodology and Computational Implementation

The predictive capacity of the framework is validated against two experimentally characterized regimes (polar liquid crystals exhibiting heliconical smectic phases [18] and flocking in metal-dielectric Janus colloids [19]) by adapting the dimensionless parameter space (Table 1) to each system. The computational implementation must satisfy the tensorial constraints of the order parameter, the stiffness of the elastic regularization, the stochastic nature of symmetry-breaking transitions, and the appropriate boundary conditions. To achieve this, a unified numerical approach is adopted, discretizing the governing equations on a uniform Cartesian grid with spacing $\Delta x^{*}$ chosen to resolve the smallest physical length scales pertinent to each regime.

The time-stepping scheme employs a second-order predictor-corrector method with adaptive time stepping. At each step $\Delta t^{*}$, the tensorial evolution Equation (20) is advanced semi-implicitly via a Crank–Nicolson scheme for the elastic diffusion term, with the implicit linear system solved using a multigrid-preconditioned conjugate gradient solver. The nonlinear terms (the bulk restoring force and active torque) are treated using second-order Adams–Bashforth extrapolation. The dynamic polar evolution Equation (21) is advanced semi-implicitly via a Crank–Nicolson scheme for its elastic diffusion term $K\nabla^{2}P$ to maintain numerical stability near critical points, the scalar equation and the momentum equation are advanced using an explicit Adams–Bashforth scheme with adaptive time-stepping, and the tensorial equation is updated using a semi-implicit scheme. The scalar field ϕ (Equation (22a)) is advanced with fully implicit diffusion and explicit Adams–Bashforth advection and active fluxes. The velocity field (Equations (22b) and (22c)) is solved using a projection method: an intermediate velocity is updated, followed by a pressure-correction step enforcing incompressibility via a Poisson equation, solved using FFT in periodic domains or multigrid in confined geometries.

Coupling between the four equations is handled through operator splitting. At each time step, the update sequence is: (i) compute the active torque and active stress; (ii) advance the macroscopic flow (iii) advance the scalar field ϕ; (iv) advance the polar field *P*; (v) advance the tensorial field *Q* using the updated fields; and (vi) recompute S* from *Q* to update the state-dependent mobility $\Gamma \left(S\right)$. This staggered approach captures all feedback pathways within each step, ensuring consistency of the complete coupled system.

Stability of the explicit advective terms is enforced via the standard Courant–Friedrichs–Lewy (CFL) condition $\Delta t^{*}<\Delta x^{*}/|U^{*}{|}_{max}$, with adaptive time stepping adjusting $\Delta t^{*}$ dynamically. Since elastic diffusion is treated implicitly, it imposes no spatial step restriction. Grids are 256–512 points in 1D ($\Delta x^{*}∼0.01$) for liquid crystals, and 512×512 points ($\Delta x^{*}∼0.02$) in 2D for colloids. Simulations start from a weakly ordered state with small fluctuations, evolve for $10^{5}$–$10^{6}$ steps to steady state, and compute order parameters by spatial averaging after discarding a $10^{4}$-step transient. The methodology is validated against analytical linearized solutions and benchmarked against independent implementations.

Grid resolution must capture the smectic layer spacing (~3 nm) and helical pitch (~hundreds of nm) for polar fluids, and the particle diameter (3 μm) and electrostatic screening length (120 μm) for Janus colloids. The dimensionless tensor $Q^{*}$ is stored as a symmetric, traceless 3×3 matrix at each grid point, retaining only its five independent components ($Q_{xx}^{*},Q_{xy}^{*},Q_{xz}^{*},Q_{yy}^{*},Q_{yz}^{*}$). The remaining components are reconstructed via symmetry $Q_{ji}^{*}=Q_{ij}^{*}$ and tracelessness $Q_{zz}^{*}=-Q_{xx}^{*}-Q_{yy}^{*}$. Although the spatial domain is reduced to 1D or 2D (eliminating out-of-plane derivatives), $Q^{*}$ retains its full 3D tensorial character to capture out-of-plane tilting and complex topologies. The scalar order parameter $S^{*}=\sqrt{\frac{3}{2}Tr\left(Q^{*2}\right)}$ is computed at each iteration, recovering the alignment magnitude for a uniaxial nematic state $Q^{*}=S^{*}\left(\hat{n}\hat{n}-\frac{1}{3}I\right)$. The polar field $P^{*}$ is stored as a three-component vector with magnitude $P^{*}=|P^{*}|$ and orientation $\hat{p}=P^{*}/P^{*}$, while the scalar control field $\phi^{*}$ is tracked as a standard scalar array.

Spatial derivatives employ a fourth-order five-point stencil for the Laplacian:

(43) $$\nabla^{*2}f_{i,j,k}^{*}=\frac{-f_{i+2,j,k}^{*}+16f_{i+1,j,k}^{*}-30f_{i,j,k}^{*}+16f_{i-1,j,k}^{*}-f_{i-2,j,k}^{*}}{12{\left(\Delta x^{*}\right)}^{2}}$$

with analogous expressions in the y- and z-directions. The vorticity tensor $\Omega_{ij}^{*}=\frac{1}{2}\left(\partial_{i}U_{j}^{*}-\partial_{j}U_{i}^{*}\right)$ is computed via matching fourth-order finite differences.

Temporal integration uses a semi-implicit Euler–Maruyama scheme: the elastic diffusion term is treated implicitly to relax the CFL constraint, while nonlinear forces and advection are explicit. The time step is bounded by the combined constraints $\Delta t^{*}\leq min(\Delta x^{*}/|U^{*}{|}_{max}De)$, where the first limit is the advective CFL condition and the second ensures resolution of the thermodynamic relaxation timescale. In practice, $\Delta t^{*}$ is set to $10^{-3}$–$10^{-4}$ of the characteristic relaxation time. The structural update follows the discretized form:

(44) $$\frac{Q^{*\left(n+1\right)}-Q^{*n}}{\Delta t^{*}}+\left[U^{*n}\cdot \nabla^{*}Q^{*n}-\Omega^{*n}\cdot Q^{*n}+Q^{*n}\cdot \Omega^{*n}\right]=\frac{1}{De}\Phi^{*}\left(F_{\mathrm{bulk}\text{-}\mathrm{restoring}}^{*},S^{*n}\right)+\frac{1}{De\sigma^{2}}\nabla^{*2}Q^{*\left(n+1\right)}+\frac{1}{De}\xi^{*n}\;$$

The Laplacian is treated implicitly (time level *n* + 1) to circumvent stiffness, while advective, active torque, and bulk restoring forces use state values at time level *n*. The implicit system is solved via a fast Poisson solver (geometric multigrid or FFT-based decomposition) for each independent component of *Q*. For steady-state solutions, temporal integration is bypassed in favor of Newton–Raphson continuation.

The Gaussian white noise $\xi^{*}$ is generated via the Euler–Maruyama method. At each grid point, independent normal random numbers with zero mean and unit variance are scaled by $\sqrt{\frac{\left(1-S^{*n2}\right)D_{\mathrm{eff}}}{\Delta t^{*}\Delta x^{*3}}}$ to satisfy Equation (31), incorporating the state-dependent jamming function to attenuate fluctuations near kinetic arrest. The symmetric, traceless noise tensor is constructed as:

(45) $$\xi_{ij}^{*n}=\frac{1}{\sqrt{2}}\left(\zeta_{ij}+\zeta_{ji}\right)-\frac{1}{3}\mathrm{Tr}\left(\zeta \right)\delta_{ij}$$

where ζ is a temporary random matrix populated with independent Gaussian entries. This construction guarantees that $\xi_{ij}^{*n}=\xi_{ji}^{*n}$, $Tr\left(\xi^{*n}\right)=0$, and that the correlation function is satisfied identically, providing the vital symmetry-breaking mechanism required to escape metastable isotropic states once critical thresholds ($\phi_{c}^{Q^{*}}$ and $\phi_{c}^{P^{*}}$) are crossed. Boundary conditions are fitted to the physical environments of each regime. For both the 1D polar liquid crystal models and the 2D Janus colloid monolayers, periodic boundary conditions are employed to capture the bulk spatiotemporal emergence of order without wall-induced interference. In the Janus colloid regime, the active flux is identified with the self-propulsion velocity field $v_{0}^{*}\hat{n}$, while the repulsive particle interactions are captured through the thermodynamic restoring force and elastic diffusion terms.

For the Janus colloid system, the scalar field ϕ represents the particle area fraction or local density. Its evolution is governed by a convection-diffusion equation that incorporates advection by the macroscopic flow, Brownian diffusion, and a pressure gradient term arising from particle interactions. This equation is a specialized form of Equation (22a) adapted to the colloidal regime. In the dimensionless form employed in the numerical simulations, the density field evolves according to:

(46) $$\frac{\partial \phi^{*}}{\partial t^{*}}+\nabla^{*}\cdot \left(\phi^{*}U^{*}\right)=\nabla^{*}\cdot \left(D\left(\phi^{*}\right)\nabla^{*}\phi^{*}\right)+\nabla^{*}\cdot \left(\mu \left(\phi^{*}\right)\phi^{*}\nabla^{*}\phi^{*}\right)-\nabla^{*}\cdot \left(\phi^{*}U_{\mathrm{active}}^{*}\right)$$

where $D\left(\phi^{*}\right)=D_{0}\left(1+\lambda_{D}\phi^{*}\right)$ is a density-dependent diffusion coefficient that captures the enhanced Brownian motion at low densities and the reduced mobility at high packing fractions, $\mu \left(\phi^{*}\right)$ is a mobility coefficient associated with the repulsive interparticle forces, and $U_{\mathrm{active}}^{*}=v_{0}^{*}\hat{n}$ is the self-propulsion velocity field of the Janus particles. The term $\nabla^{*}\cdot \left(\mu \left(\phi^{*}\right)\phi^{*}\nabla^{*}\phi^{*}\right)$ represents the particle pressure gradient, where $\mu \left(\phi^{*}\right)$ encodes the dependence of the effective pressure on the local density through the virial expansion of the equation of state. This pressure term arises from the variational derivative of the interaction free energy $\delta F_{\mathrm{int}}/\delta \phi$, where $F_{\mathrm{int}}$ captures the purely repulsive excluded-volume interactions between the colloids. The active flux term $-\nabla^{*}\cdot \left(\phi^{*}U_{\mathrm{active}}^{*}\right)$ describes the advection of particles by their own self-propulsion, which, when combined with the turn-away torques that reorient particles upon collision, gives rise to the macroscopic flocking transition observed experimentally.

The coupling between the density field and the structural order parameters is established through the dependence of the free energy coefficients on $\phi^{*}$. Specifically, the tensorial and polar instability thresholds are modulated by the density according to $A_{Q}\left(\phi^{*}\right)=a_{Q}\left(\phi^{*}-\phi_{c}^{Q^{*}}\right)$ and $A_{P}\left(\phi^{*}\right)=a_{P}\left(\phi^{*}-\phi_{c}^{P^{*}}\right)$, where $\phi_{c}^{Q^{*}}$ and $\phi_{c}^{P^{*}}$ represent the critical area fractions for the onset of nematic and polar order, respectively. This density dependence captures the physical observation that increasing the particle concentration enhances the collision frequency and promotes collective alignment, while simultaneously increasing the excluded-volume constraints that attenuate the tensor-polar coupling through the exponential factor $\gamma \left(S\right)=\gamma_{0}e^{-\lambda S}$.

### 3.3. Polar Liquid States and Heliconical Smectic Phases

Consider the experimental observations of Gibb et al. [18], who studied two novel polar liquid phases: the polar heliconical smectic C (${\mathrm{SmC}}_{P}^{H}$) phase and the antiferroelectric orthogonal smectic A (AFSmA) phase. These phases emerge from a cascade of symmetry-breaking transitions in compounds with large electric dipole moments, providing a direct test of the sequential symmetry-breaking mechanism contained in the dimensionless parameters of our formulation. The experimental system of compound **1** (ref. [18], Figures 2 and 3) exhibits the following phase sequence upon cooling from the isotropic liquid: isotropic to nematic (N) at 154.3 °C, nematic to SmA at 129.7 °C, SmA to ferroelectric smectic A (${\mathrm{SmA}}_{F}$) at 111 °C, and ${\mathrm{SmA}}_{F}$ to ${\mathrm{SmC}}_{P}^{H}$ at 90 °C.

The primary tensorial transition occurs when the scalar field $\phi^{*}$, representing the reduced temperature, crosses the primary critical threshold $\phi_{c}^{Q*}$. At this thermodynamic coordinate, the quadratic coefficient $A_{Q}\left(\phi^{*}\right)=a_{Q}\left(\phi^{*}-\phi_{c}^{Q*}\right)$ becomes negative, and the isotropic state $Q^{*}=0$ becomes linearly unstable, causing the system to spontaneously develop nematic order corresponding to the N phase observed experimentally (ref. [18], Figure 2a). The development of smectic layering is captured by the spatial regularization term $\frac{1}{\sigma^{2}}\nabla^{*2}Q^{*}$ and the scalar field coupling. When the correlation length $\xi =\sqrt{L/A_{0}}$ becomes comparable to the molecular length such that the confinement parameter matches the geometric ratio $\sigma ∼l_{0}/\xi$, the system undergoes a transition to a lamellar structure corresponding to the SmA phase.

Upon further cooling, $\phi^{*}$ crosses the secondary threshold $\phi_{c}^{P*}$, at which point $A_{P}\left(\phi^{*}\right)=a_{P}\left(\phi^{*}-\phi_{c}^{P*}\right)$ becomes negative and the polar vector field $P^{*}$ spontaneously emerges. This activates the internal torque ${\left(P^{*}⊗P^{*}\right)}^{ST}$ in the dimensionless evolution equation, driving the transition to the polar ${\mathrm{SmA}}_{F}$ phase (ref. [18], Figure 2b). When the dimensionless active torque number exceeds the critical threshold discussed before, the uniform polar state becomes unstable and the system dynamically alter its internal architecture to minimize the bulk electrostatic energy. In the ${\mathrm{SmA}}_{F}$ phase, the polar vector $P^{*}$ is aligned along the layer normal, generating a macroscopic polarization whose resulting electrostatic energy penalty scales as $\epsilon^{2}$. To escape this penalty, the system spontaneously develops spatial modulations, leading to either the ${\mathrm{SmC}}_{P}^{H}$ phase characterized by continuous helical precession or the AFSmA phase characterized by a discrete block structure (ref. [18], Figures 2c and 4).

For the polar fluid simulations, as De→0 corresponding to the kinematically stationary limit with $U^{*}=0$, and neglecting time dependence and fluctuations, the generalized dimensionless Equation (24) reduces to the steady-state torque balance:

(47) $$\epsilon {\left(P^{*}⊗P^{*}\right)}^{ST}-\frac{\delta F_{\mathrm{bulk}}^{*}}{\delta Q^{*}}=0$$

with the emergent polar vector $P^{*}$ acting as the internal active flux replacing the external drive $J^{*}$, and the bulk free energy evaluated from Equation (28).

The dimensionless parameters are estimated from the experimental data of Gibb et al. [18]: $\phi^{*}$ ranges from 0.1 to 1.0 as the reduced temperature, with $\phi^{*}=1.0$ at the SmA–${\mathrm{SmA}}_{F}$ transition; $\phi_{c}^{Q*}=0.8$ indicates the onset of nematic order at 154.3 °C; $\phi_{c}^{P*}=0.5$ indicates the onset of polar order at 111 °C; ϵ ranges from 1 to 5 as the active torque from molecular dipoles varies with temperature; σ ranges from 10 to 100 as the ratio of cell thickness (5 μm) to correlation length (50 nm); b ranges from 0.5 to 1.0 as the first-order transition parameter; $g_{0}$ ranges from 0.5 to 2.0 as the baseline tensor-polar coupling estimated from Second Harmonic Generation (SHG) intensity; and the high-order coefficients ($e,e_{P}>0$) provide the necessary steric capping at high activity. The calculations are performed on a 1D grid along the helical axis with periodic boundary conditions and a grid spacing of $\Delta x^{*}=0.01$ in reduced units. The steady-state solutions are obtained by solving the coupled equations for $Q^{*}$ and $P^{*}$ using a Newton–Raphson method with continuation in the temperature parameter $\phi^{*}$.

The simulated phase sequence is shown in Figure 1a–c. At high temperatures ($\phi^{*}>\phi_{c}^{Q*}$), the isotropic state is stable with $Q^{*}=0$ and $P^{*}=0$. As $\phi_{0}^{*}$ decreases below $\phi_{c}^{Q*}$, the system develops uniaxial nematic order with $S^{*}>0$ but $P^{*}=0$, corresponding to the apolar nematic phase. Further cooling below $\phi_{c}^{P*}$ triggers the emergence of polar order, with $P^{*}$ growing continuously from zero in a second-order transition, while $S^{*}$ continues to increase. This sequential symmetry-breaking cascade precisely mirrors the experimental observations of Gibb et al. [18] at Figure 3, including the temperature dependence of the second- and fourth-rank orientational order parameters $\left\langle P_{2}\right\rangle$ and $\left\langle P_{4}\right\rangle$ obtained by polarized Raman scattering.

For compound **4** (ref. [18], Figure 4), which exhibits the AFSmA phase, the simulation predicts a 1D modulated structure of alternating polar blocks. The scalar order parameter $S^{*}$ oscillates periodically along the spatial coordinate, with regions of positive $S^{*}$ representing polar blocks with $P^{*}$ aligned along $+\hat{z}$ and negative $S^{*}$ representing blocks with $P^{*}$ aligned along $-\hat{z}$. The block periodicity p is determined by the balance between the active torque ϵ and the confinement parameter σ according to:

(48) $$p∼\frac{2\pi \sigma }{\sqrt{\epsilon -\epsilon_{c}}}$$

which is derived directly from the linear stability analysis of the uniform polar state. For the experimental parameters, this gives $p∼10^{2}\;\mathrm{nm}$, consistent with the observed block structure. The antiferroelectric nature is confirmed by the absence of a net macroscopic polarization ($\left\langle P^{*}\right\rangle =0$) when averaged over a full spatial period, in agreement with the experimental observation of a double-peaked current response (ref. [18], Figure 4a) and the complete absence of an SHG signal.

For compound **1**, the simulation predicts a different modulation, a continuous helical precession of the tilt direction orthogonal to the layer normal. The polar vector $P^{*}$ tilts relative to the layer normal, and the local azimuthal tilt angle θ oscillates sinusoidally along the spatial coordinate according to:

(49) $$\theta \left(x\right)=\theta_{0}\sin\left(\frac{2\pi x}{\lambda_{\mathrm{pitch}}}\right)$$

where the characteristic helical pitch $\lambda_{\mathrm{pitch}}$ is given analytically by:

(50) $$\lambda_{\mathrm{pitch}}∼\frac{2\pi \sigma }{\sqrt{g_{0}\epsilon -1}}$$

This equation shows that when $g_{0}\epsilon <1$, the uniform state remains stable and no modulation occurs, but when $g_{0}\epsilon >1$, the uniform phase becomes unstable, forcing the transition to the modulated helical state. The −1 term represents the critical threshold where active driving overcomes the stabilizing elastic contributions. For the experimental parameters ($\sigma ∼50,\;g_{0}∼1,\epsilon ∼2$), this yields $\lambda_{\mathrm{pitch}}∼10^{3}\;\mathrm{nm}$, consistent with the observed selective reflection of visible light (ref. [18], Figure 2g). The helical precession is accompanied by a continuous variation in the optical birefringence which agree with the striated texture observed experimentally (ref. [18], Figure 2c). The predicted relationship between the helical pitch and temperature, $\lambda_{\mathrm{pitch}}\left(T\right)\propto {\left[g_{0}\epsilon \left(T\right)-1\right]}^{-1/2}$, is consistent with the slight temperature dependence observed in the experimental spectroscopy (ref. [18], Figure S18).

The simulated histogram of the azimuthal phase angle ψ for the ${\mathrm{SmC}}_{P}^{H}$ phase, shown in Figure 1d, exhibits two symmetric peaks separated by π radians, representing the 50-50 split in the molecular tilt orientation. This is a clear signature of spontaneous symmetry breaking, where the continuous $U\left(1\right)$ symmetry of the uniform state is reduced to a discrete $Z_{2}$ symmetry. The statistical distribution is in agreement with the experimental histogram reported by Gibb et al. [18] at Figure S22, including the equal populations of the two-phase states with p=0.50±0.02.

The simulated 3D phase diagram shown in Figure 2 maps the structural geometries onto the dimensionless parameter space $\left(\phi^{*},1/\sigma ,\epsilon \right)$. The diagram is constructed by varying the dimensionless parameters and determining the ground-state structure via energy minimization of the expanded free energy functional. The topological boundaries are governed by the close interplay between the composition-dependent scalar field, the spatial confinement ratio, and the dimensionless active torque number.

The critical boundary corresponds to the thermodynamic threshold where the dimensionless ratio $\epsilon /\sigma^{2}$ crosses unity, forcing the spontaneous emergence of modulated phases. When $\epsilon <\sigma^{2}$, the elastic energy cost of spatial gradients exceeds the benefit of escaping the bulk polar order, keeping the uniform state stable. When $\epsilon >\sigma^{2}$, the uniform state becomes unstable, and the system spontaneously develops spatial modulations.

The boundary between the ${\mathrm{SmC}}_{P}^{H}$ and AFSmA phases is determined by the balance between the coupling constant $g_{0}$ and the tilt elastic constant. For large $g_{0}$ corresponding to strong $Q^{*}$– $P^{*}$ coupling, the system presents the continuous helical precession of the ${\mathrm{SmC}}_{P}^{H}$ phase, while for small $g_{0}$, the discrete block structure of the AFSmA phase is favored. This phase boundary is located at $g_{0}∼1/\epsilon$, consistent with the experimental observation that compound **1** (large $g_{0}$) exhibits the ${\mathrm{SmC}}_{P}^{H}$ phase, while compound **4** (small $g_{0}$) exhibits the AFSmA phase. The binary mixture phase diagram (ref. [18], Figure 5a) is reproduced by mapping the composition onto the scalar field $\phi^{*}$. As the concentration of DIO increases, $\phi^{*}$ increases, corresponding to an effective shift in transition boundaries that allows the room-temperature stabilization of the $N_{F}$ and ${\mathrm{SmC}}_{P}^{H}$ phases observed experimentally for mixtures with 35–65% DIO.

The quantitative comparison with experimental data yields agreement across all measured quantities. The simulated tilt angle θ as a function of temperature, obtained from the polar vector orientation, increases from 0° at the ${\mathrm{SmA}}_{F}$–${\mathrm{SmC}}_{P}^{H}$ transition to a maximum of approximately 23° at lower temperatures, in agreement with the SAXS measurements of Gibb et al. [18] at Figure 3a. The temperature dependence follows the mean-field scaling:

(51) $$\theta \left(T\right)\propto \sqrt{\phi_{c}^{P*}-\phi^{*}\left(T\right)}$$

consistent with the second-order nature of the transition with a critical exponent of β=1/2. The simulated layer spacing $d\left(T\right)$ is temperature-independent in the ${\mathrm{SmA}}_{F}$ phase with d∼3 nm, showing a small decrease upon entering the ${\mathrm{SmC}}_{P}^{H}$ phase due to the molecular tilt according to $d\left(T\right)=d_{0}\cos\theta \left(T\right)$, in agreement with the experimental SAXS data (ref. [18], Figure 3a).

The simulated second harmonic generation intensity scales as $I_{\mathrm{SHG}}\propto {\left|P^{*}\right|}^{2}$ and predicts an increase at the SmA–${\mathrm{SmA}}_{F}$ transition, followed by a plateau in the ${\mathrm{SmA}}_{F}$ phase and a further increase in the ${\mathrm{SmC}}_{P}^{H}$ phase, in agreement with the experimental SHG measurements (ref. [18], Figure 3d). The simulated spontaneous polarization $P_{s}$ increases continuously from zero at the SmA–${\mathrm{SmA}}_{F}$ transition, following the mean-field scaling:

(52) $$P_{s}\left(T\right)\propto \sqrt{\phi_{c}^{P*}-\phi^{*}\left(T\right)}$$

reaching values of approximately $1-2\;\mu C/{\mathrm{cm}}^{2}$ in the ${\mathrm{SmA}}_{F}$ and ${\mathrm{SmC}}_{P}^{H}$ phases, consistent with the experimental measurements for compounds **2**–**4** (ref. [18], in their Figures 4b and S14). This square-root dependence near the bifurcation point is characteristic of second-order phase transitions with a critical exponent of β=1/2, matching the mean-field scaling observed experimentally. This agreement across multiple experimental observables confirms the predictive capacity of our framework for polar liquid crystalline phases and validates the sequential symmetry-breaking mechanism contained in the dimensionless parameters.

### 3.4. Flocking in Metal-Dielectric Janus Colloids

Consider now the experimental observations of Das et al. [19], who demonstrated that self-propelled Janus colloidal particles can exhibit spontaneous flocking despite interacting through repulsive forces and turn-away torques that intuitively seem to prevent polar order. This system provides a distinct test of the framework’s capacity to describe the emergence of macroscopic polar order from microscopic interactions that do not implement polar alignment, thereby bridging the classes of alignment-based and repulsion-based active matter. The experimental system (ref. [19], Figure 1) consists of 3-μm-diameter silica spheres coated with 35 nm of titanium and 20 nm of silicon oxide on one hemisphere. These particles are suspended in deionized water and confined between conductive coverslips separated by a 120 μm spacer. Upon sedimentation, the particles form a monolayer on the bottom coverslip. The application of a perpendicular AC voltage of amplitude 10 V and frequency 30 kHz aligns the particle equator perpendicular to the coverslips and polarizes the metal and dielectric hemispheres differently, inducing particle self-propulsion along a direction pointing from the dielectric to the metallic hemisphere, as well as electrostatic interparticle forces and torques (ref. [19], Figure 1b). The system remains as an isotropic active gas at low area fractions and self-propulsion speeds (ref. [19], Figure 1c), but develops polar order and flocks at higher area fractions and speeds, with spatiotemporal patterns including vortices and large-scale polar bands characteristic of flocking systems (ref. [19], in their Figure 1d, Movies 2–4).

The key microscopic interactions in this system are the repulsive force between particles:

(53) $$F_{ij}=\left[\frac{3{\left(d_{h}+d_{t}\right)}^{2}}{4\pi \epsilon_{s}}\right]\frac{e^{-r_{ij}/\lambda }}{r_{ij}^{4}}{\hat{r}}_{ij},$$

where $\epsilon_{s}$ is the dielectric permittivity of the solvent, $d_{h}>0$ and $d_{t}<0$ are the effective dipole strengths of the head and tail hemispheres, and the exponential factor accounts for screening by the electrodes separated by a distance λ=120 μm. The particles also interact via a turn-away torque:

(54) $$\Gamma_{ij}=\left[\frac{3l\left(d_{h}^{2}-d_{t}^{2}\right)}{4\pi \epsilon_{s}}\right]\frac{e^{-r_{ij}/\lambda }}{r_{ij}^{4}}\left({\hat{n}}_{j}\times {\hat{r}}_{ij}\right),$$

where l=3R/8 is the distance by which the dipoles are off-centred, with R=1.5 μm the particle radius (ref. [19], Equation (2)). The torque tends to turn a particle with orientation ${\hat{n}}_{j}$ away from the interparticle distance vector ${\hat{r}}_{ij}$. These turn-away interactions are fundamentally different from Vicsek-type alignment interactions. Whereas alignment interactions couple the orientations of two particles ($\Gamma_{ij}\propto {\hat{n}}_{i}\times {\hat{n}}_{j}$), the turn-away interactions couple the orientation of one particle to the position of another ($\Gamma_{ij}\propto {\hat{n}}_{j}\times {\hat{r}}_{ij}$), making them intrinsically non-reciprocal: $\Gamma_{ij}\neq -\Gamma_{ji}$ (ref. [19], Equation (2)).

The active flux in this system corresponds to the self-propulsion velocity of the particles, $J_{0}=v_{0}{\hat{n}}_{i}$, which drives the system out of equilibrium. The key dimensionless parameters governing the Janus colloid dynamics can be mapped onto our framework’s dimensionless numbers. The active torque number, $\epsilon =\alpha J_{0}^{2}/\left(A_{0}Q_{0}\right)$, scales quadratically with velocity ($\epsilon \propto v_{0}^{2}$) since the active kinetic energy flux satisfies $J_{0}^{2}∼v_{0}^{2}$. This number also depends on the local area fraction $\phi_{0}$, quantifying the competition between active driving and thermodynamic restoring forces.

The confinement parameter, $\sigma =l_{0}\sqrt{A_{0}/L}$, compares the system size to the correlation length. In the Janus system, σ is controlled by the screening length λ=120 μm and the characteristic particle interaction range. The scalar Péclet number, $Pe_{\phi }=U_{0}l_{0}/\left(M_{\phi }A_{0}\right)$, compares advective transport to the diffusive relaxation of the scalar field. The Ericksen number, $Er=\frac{U_{0}l_{0}}{\Gamma_{0}L}$ quantifies the competition between advection and elastic diffusion, while the Deborah number, $De=\frac{\tau U_{0}}{l_{0}\Gamma_{0}A_{0}}$, characterizes the structural memory of the system relative to its baseline relaxation rate. The coupling parameters b, e, $e_{P}$, and $g_{0}$ are material-dependent and control the first-order character of the transition, the steric saturation, and the coupling between tensorial and polar order, respectively.

For the Janus colloid simulations, we consider the dimensionless tensorial evolution equation given by Equation (24) coupled directly to the scalar transport equation. The active torque term $\epsilon {\left(J^{*}⊗J^{*}\right)}^{ST}$ seamlessly captures the turn-away interactions, the active flux J is identified with the self-propulsion velocity field $v_{0}^{*}\hat{n}$, and the symmetric-traceless projection encodes the alignment of the tensorial order parameter with the direction of motion. The repulsive forces between particles are captured through the bulk free energy gradient, $-\frac{\delta F_{bulk}^{*}}{\delta Q^{*}}+\frac{1}{\sigma^{2}}\nabla^{*2}Q^{*}$, which penalizes spatial variations and balances order parameter magnitudes.

The scalar field $\phi^{*}$ represents the local density or area fraction of particles, and its evolution is governed by the dimensionless scalar transport equation. The particles are advected by the flow and subject to Brownian motion, with the effective repulsive pressure $F_{rep}\propto \nabla^{*}\phi^{*}$ capturing the interactions arising from the density gradient. The simulations are performed on a 2D grid in the plane of the particle monolayer with periodic boundary conditions, a grid spacing of $\Delta x^{*}=0.01$ in reduced units, and a time step of $\Delta t^{*}=10^{-4}$. The initial state is a uniform, isotropic distribution of particles with random orientations, corresponding to the isotropic gas phase observed experimentally at low area fractions and self-propulsion speeds (ref. [19], Figure 1c). The scalar order parameter, $S^{*}=\sqrt{\frac{3}{2}Tr\left(Q^{*2}\right)}$, measures the local degree of structural alignment.

Figure 3a shows the primary bifurcation at $\epsilon_{c}=1$ separating the isotropic gas regime from the polar flocking regime. The experimental data points from Das et al. [19] at Figure 1e, are shown as grey circles for the isotropic gas phase, red squares for the transition boundary, and blue triangles for the polar flocking phase, with the predicted phase boundary in agreement with the experimental results. This shows that the active torque number serves as a bifurcation parameter governing the onset of polar order in systems with turn-away interactions.

The continuous nature of the flocking transition and the relationship between tensorial and polar order are validated by the scalar and polar order parameters shown in Figure 3b. The polar order parameter $P=\left\langle \left|\Sigma_{i}{\hat{n}}_{i}\right|/N\right\rangle$ and the scalar order parameter $S^{*}$ are obtained from simulations of Equation (24). Near the threshold, the polar order parameter matches the standard Landau expansion:

(55) $$P=\sqrt{\frac{a}{b_{3}}},$$

where $a=\rho \xi \left(\epsilon -\epsilon_{c}\right)-D_{r}$ is the linear growth rate derived from the BBGKY coarse-graining and $b_{3}$ is the third-order Landau coefficient. Since $a\propto \epsilon -\epsilon_{c}$ near the transition, this yields the square-root dependence $P\propto \sqrt{\epsilon -\epsilon_{c}}$ near the threshold (ϵ≈1), which is characteristic of second-order phase transitions with a mean-field critical exponent of β=1/2. The fitted curve with parameters ρξ=0.321, $D_{r}=0.108{\;s}^{-1}$, and $b_{3}=0.523$ reproduces the experimental data near the onset of order. Notice that the continuous growth of both order parameters from zero at the transition confirms the second-order nature of the flocking transition and validates the predictive capacity of the tensorial field theory. This is consistent with the Boltzmann kinetic theory predictions of Das et al. [19] at Equation (7) and Equation (A4), which yield $P=\sqrt{a/b}$ with $a\propto \epsilon -\epsilon_{c}$ and b>0, also indicating a second-order transition with a critical exponent of β=1/2.

The microscopic mechanism responsible for flocking can be understood through the coarse-graining of the microscopic model via the BBGKY hierarchy (ref. [19], Appendix B), which yields a hydrodynamic equation for the polarity field of the form $\partial_{t^{*}}P^{*}=a\left[\rho \right]P^{*}+O\left(\nabla^{*}\right)$, with the growth rate given by $a\left[\rho \right]=\frac{\rho \tau_{0}}{2\pi \xi_{r}}-D_{r}$ (ref. [19], Equation (5)). This is the form predicted by the present framework in the limit where the tensorial order parameter is enslaved to the polar field, with the growth rate mapping onto the active torque number $a∼\epsilon -\epsilon_{c}$. The coefficient $\tau_{0}$, which captures the effect of the turn-away torques through the pair distribution function and is given by an integral over the pair distribution function $g\left(r,\phi ,\theta \right)$ (ref. [19], Equation (6)), corresponds to the active torque $\alpha {\left(J⊗J\right)}^{ST}$ in the present framework, while the rotational diffusion coefficient $D_{r}$ corresponds to the thermodynamic restoring force.

As demonstrated by Das et al. [19] at Figure 4b–e, the pair distribution function in the isotropic state exhibits the four symmetry-breaking conditions required for a non-zero $\tau_{0}$, asymmetry upon changing the sign of the polar angle, upon changing the sign of the relative orientation, and upon the transformations ϕ→ϕ+π and θ→θ+π. These asymmetries arise from the combined effects of turn-away torques, which create low-torque regions where particles tend to stay longer, and repulsive forces, which produce front-back asymmetries in the particle distribution (ref. [19], Figure 4h,i). These symmetry-breaking correlations are captured by this formulation through the active torque term and the thermodynamic restoring force.

The phase diagram in Figure 4 maps the full behavior of the Janus colloid system onto the three-dimensional dimensionless parameter space of the tensorial field theory. The three axes represent the active torque number, the inverse confinement parameter, and the scalar field representing the area fraction. The primary bifurcation surface at $\epsilon_{c}=1$ separates the isotropic gas from the polar flocking regimes. The secondary boundaries at $\phi^{*}≃0.5$ and $\phi^{*}≃0.6$, derived from the experimental observations of Das et al. [19] at Figure 3a, separate the fluid flocks from the hexatic and crystalline phases, respectively. The confinement parameter controls the elastic cost of spatial gradients. For small 1/σ (strong confinement), the system is locked into the uniform polar state, while for large 1/σ (weak confinement), modulated and crystalline structures emerge.

The predictive capacity of the framework is further accounted by the observation of active Wigner crystals at high area fractions (ref. [19], Figure 3). As the area fraction increases beyond the flocking threshold, the system develops crystalline order while maintaining polar order, forming active Wigner crystals, a phenomenon proposed for electron gases and here realized in an active colloidal system through purely repulsive interactions combined with turn-away torques (ref. [19], Figure 3). In the present framework, this behavior is captured by the competition between the active torque and the confinement parameter. At high area fractions, the scalar field $\phi^{*}$ becomes large, increasing the confinement σ and the elastic energy cost of spatial gradients. The system responds by forming crystalline order to minimise the free energy, while the polar order is maintained by the active torque.

The agreement between the predicted phase boundaries and the experimental observations demonstrates that this formulation provides a generalized description of the isotropic gas, fluid flocks, hexatic flocks, and active Wigner crystals observed in the Janus colloid system, with the active torque number emerging as the universal driver of structural bifurcation across all regimes. This quantitative agreement across multiple independent experimental observables (the sharp transition at critical area fraction and self-propulsion speed captured in Figure 3a, the continuous growth of polar order from zero with the characteristic $P\propto \sqrt{\epsilon -\epsilon_{c}}$ scaling near the critical threshold, and the saturation of both order parameters at high active torque captured by the state-dependent mobility and 6th-order free energy terms (Figure 3b) demonstrates that the phenomenological framework captures the complex active phenomena in metal-dielectric Janus colloids and validates the active torque number as the universal bifurcation parameter governing the transition from isotropic disorder to polar order.

## 4. Comparative Analysis with Other Theoretical Frameworks

The characteristics and scope of the present phenomenological framework are better understood when compared against established theoretical approaches that have shaped our understanding of symmetry-breaking phenomena in liquid crystals and complex fluids. Specifically, we contrast our formulation with the mesoscopic Chen–Lubensky model [29] and classical Molecular Field Theories [22,30], highlighting how a shift toward non-equilibrium, high-order kinematics fundamentally changes our capacity to describe strongly driven soft matter.

The Chen–Lubensky model [29] operates as a landmark higher-order extension of the classical Landau–de Gennes framework [22], developed primarily to map the interplay within smectic liquid crystals. As a mesoscopic mean-field theory, it focuses on the direct coupling between a coarse-grained molecular director field and a complex smectic order parameter that characterizes the periodic density modulations of layered phases. This structural coupling enabled the model to achieve remarkable predictive success across several historically challenging domains. Most notably, it predicted the existence of twist-grain-boundary phases, where smectic layers systematically twist relative to one another by organizing into regular lattices of screw dislocations. Furthermore, the model has been utilized to solve localized director and layer profiles in surface-stabilized smectic-C cells (capturing the electric-field-induced “melting” of layers near chevron tips) and to describe the Helfrich-Hurault effect, where external magnetic or electrical fields trigger periodic layer undulations in smectic-A matrices. Despite this, the Chen–Lubensky model [29] exhibits fundamental structural limitations when applied to highly driven, far-from-equilibrium active matter. Because it leaves out short-range isotropic entropy contributions, the model is not capable of self-consistently tracking the isotropic-to-nematic transition initiating from a high-temperature state. More critically, it is a quiescent equilibrium theory designed to map static free energy landscapes and isolate their corresponding minimizers. It lacks a fundamental, self-consistent dynamical evolution equation for its primary order parameters. Consequently, the framework cannot accommodate non-equilibrium driving forces, continuous active energy injection, or flux-driven symmetry-breaking cascades. It focuses almost exclusively on the direct interaction between an apolar nematic director and geometric layering, meaning it cannot describe the full tensorial nature of biaxial order, nor can it track the hierarchical emergence of polar vector fields within a single hydrodynamic continuum. Finally, its practical execution demands an expansive, highly specialized suite of elastic constants, layer compression moduli, and cross-coupling coefficients, many of which must be extracted empirically for each individual compound.

Molecular Field Theories [22,30] serve as an indispensable cornerstone in the physics of structured complex fluids by simplifying many-body intermolecular interactions into a manageable, self-consistent mean-field approximation. By replacing microscopic particle–particle forces with an effective average structural environment, these theories provide a highly capable tool for calculating macro-scale behaviors. They excel at mapping complex phase diagrams, predicting clearing temperatures, and isolating orientational order parameters. In recent years, they have proven vital as an initial approach for bridging the microscopic properties of ferroelectric nematics (such as permanent molecular dipoles and anisotropic polarizabilities) to macroscopic thermodynamic observables like spontaneous polarization. Additionally, by accounting for both second-rank and fourth-rank orientational averages, molecular field models successfully reproduce the temperature dependence of orientational alignment, with modern variations integrating dipole–dipole tensors, chiral geometries, and odd-elastic corrections to better capture tight molecular constraints. Nevertheless, the core assumptions underlying Molecular Field Theories [22,30] impose systematic limitations when moving far from equilibrium. Because they rely entirely on a localized mean-field approximation, they completely neglect spatial correlations and critical fluctuations, making them inherently less reliable near second-order phase transitions or critical coordinates. Furthermore, because their foundation is rooted in equilibrium thermodynamics [21], they are unable to describe true hydrodynamic transport, non-equilibrium advection, or the emergence of collective flocking patterns.

The predictive accuracy of these models remains deeply dependent on the availability of precise microscopic parameter data, such as orientation-dependent pair potentials, which are notoriously difficult to isolate experimentally. Because they are primarily tailored to describe spatially homogeneous bulk fluids, they cannot capture complex spatial pattern formation, modulated boundaries, or the dynamics of moving topological defects. Their fundamental reliance on the classical fluctuation-dissipation theorem makes them incompatible with active fluids that continuously inject energy at the individual constituent scale.

## 5. Generalized Thermodynamic-Kinematic and Framework Limitations

The formulation developed in this study offers a generalized thermodynamic-kinematic synthesis that builds upon and extends the equilibrium-based Chen–Lubensky model [29] and classical molecular field frameworks [22,30]. By incorporating non-equilibrium driving and state-dependent mobility, the present approach aims to complement these established theories, particularly for systems operating far from thermodynamic equilibrium. This section summarizes the key innovations of the framework and provides a balanced discussion of its underlying assumptions, inherent approximations, and scope of applicability.

### 5.1. Thermodynamic-Kinematic Synthesis and Core Contributions

A central feature of this formulation is the use of a single, symmetric, traceless second-rank tensor $Q\left(r,t\right)$ as the foundational mesoscopic structural descriptor. This tensorial representation describes both uniaxial and biaxial fields self-consistently, without requiring separate director vectors or ad-hoc coupling prescriptions. Departing from static equilibrium assumptions, the framework draws on recent developments in non-equilibrium thermodynamics [21]. In particular, it extends linear Onsager relations by introducing a state-dependent jamming mobility defined by the nonlinear operator $\Gamma \left(S\right)=\Gamma_{0}\left(1-S^{2}/S_{max}^{2}\right)$. This kinematic nonlinearity seeks to capture the behavior of dense, highly driven active media: as constituents align and pack into dense flocking bands, the available free volume diminishes, leading to localized structural arrest that reduces the system’s capacity to further deform or relax under active stresses.

This kinematic saturation is complemented by corresponding advances in the bulk free energy expansion. To represent excluded-volume and packing constraints in crowded environments, sixth-order steric penalties scaled by positive coefficients *E* and *E_P_* are incorporated. These terms introduce a steep energetic penalty that prevents the tensor and vector order parameters from diverging at elevated active driving forces, providing a mathematical bound consistent with physical packing limits [19]. In addition, the framework employs an order-dependent tensor-polar coupling coefficient $\gamma \left(S\right)=\gamma_{0}e^{-\lambda S}$, which decays exponentially with increasing alignment. This functional form reflects a physically motivated mechanism: in highly crowded, intensely driven states, the local collision frequency increases and strong microscopic turn-away torques emerge, reducing the efficiency with which the tensorial scaffold can induce or maintain alignment with the emergent polar vector field.

The hierarchical interplay of these fields is mediated by a conserved scalar field $\phi \left(r,t\right)$, which serves as a generalized control parameter that modulates the instability thresholds of both the tensorial and polar potentials. By defining critical coordinates $\phi_{c}^{Q}$ and $\phi_{c}^{P}$ with $\phi_{c}^{Q}>\phi_{c}^{P}$, the framework yields a sequential symmetry-breaking cascade wherein apolar nematic order stabilizes first, followed by a secondary bifurcation to polar vector order. To drive the continuum out of equilibrium, the framework introduces the dimensionless active torque number ϵ, which quantifies the intensity of non-equilibrium energy injection relative to bulk restoring stresses. When active driving exceeds localized dissipation ($\epsilon >\epsilon_{c}≃1$), the quiescent system undergoes a dynamical bifurcation, transitioning spontaneously into self-organized convective loops or large-scale flocking bands. Frame invariance throughout these highly advective macroflows is maintained by updating all structural fields through the objective corotational Jaumann rate $\dot{Q}$, while a closed feedback loop couples structural evolution back to scalar transport, driving uphill diffusion and localized mass accumulation.

A distinctive feature of this framework, compared with traditional liquid crystal models that often rely on decoupled cross-interactions or detailed molecular parameters, is its dimensional unification. The entire far-from-equilibrium state space is reduced to a minimal set of independent dimensionless groups. The Deborah number quantifies the structural memory of the medium relative to thermodynamic relaxation timescales, while the active torque number tracks the magnitude of non-equilibrium energy injection. Spatial penalties are captured by the confinement parameter, which balances macroscopic system boundaries against internal correlation lengths, and the remaining material properties are scaled by the steric and coupling groups. This unified parameterization enables quantitative comparisons across physically disparate systems. By incorporating the state-dependent jamming mobility into the generalized fluctuation-dissipation relation, the framework simultaneously describes stochastic, fluctuation-induced symmetry breaking. As shown in Section 3, the evolution equation captures both the continuous helical precession characterizing polar smectic liquid crystals [18] and the large-scale flocking bands emerging in purely repulsive Janus colloid monolayers [19].

### 5.2. Key Assumptions, Approximations, and Scope of Applicability

A balanced assessment of any theoretical framework requires a clear articulation of its underlying assumptions, inherent approximations, and the boundaries of its applicability. The present formulation, while offering a unified description of symmetry-breaking transitions in driven complex fluids, is subject to several limitations that must be acknowledged and understood. These limitations do not invalidate the framework’s utility; they define clear pathways for future development and generalization. By articulating the assumptions and approximations inherent in the present formulation, we aim to provide a realistic assessment of the framework’s scope of applicability and to guide future extensions of the formulation.

The first and most fundamental assumption is the phenomenological nature of the free energy expansion. The specific polynomial forms in Equations (12)–(14) represent a controlled Taylor expansion of the free energy density in the order parameters about the isotropic reference state. While this is the standard and most general approach within the Landau–de Gennes paradigm for continuous and weakly first-order transitions, it may not capture the full complexity of the free energy landscape in systems with strong fluctuations, long-range interactions, or non-analytic dependencies. The framework is modular in the sense that these functional forms can be replaced or extended, but the polynomial expansion does impose a smoothness assumption on the free energy that may break down near critical points or in strongly inhomogeneous configurations. For active systems where the departure from equilibrium is extreme, the validity of a free energy functional derived from equilibrium thermodynamics is an approximation that may require generalization to fully non-equilibrium thermodynamic potentials.

The second limitation concerns the state-dependent jamming mobility $\Gamma \left(S\right)=\Gamma_{0}\left(1-S^{2}/S_{\max}^{2}\right)$. This closure, while capturing the physical observation of kinetic arrest in densely packed systems, is phenomenological in origin and assumes a specific functional dependence of the mobility on the scalar order parameter. Alternative forms for the mobility function, such as or $\Gamma \left(S\right)=\Gamma_{0}e^{-\lambda S^{2}}$, may describe different systems or regimes. The framework accommodates such alternatives through the modular flux–force function $\Phi^{*}\left(F^{*},S^{*}\right)$, but the specific choice of the jamming function is material-dependent and requires validation against experimental data for each system under investigation.

The third limitation is the slaving approximation for the polar vector field *P*, in which the polar order is treated as enslaved to the tensorial order parameter through the local algebraic relation $\partial f_{\mathrm{bulk}}/\partial P=0$. This approximation assumes that the polar relaxation time is shorter than the tensorial relaxation time, enabling the adiabatic elimination of the fast polar dynamics. While our formulation incorporates a dynamical evolution equation for the polar field to accurately capture the divergence of relaxation times near the continuous phase transition threshold $\phi_{c}^{P}$, the ratio of the polar relaxation time $\tau_{P}$ to the tensorial relaxation time $\tau_{Q}$ remains a material-specific parameter. In regimes where $\tau_{P}\ll \tau_{Q}$, the system approaches the adiabatic limit, whereas comparable timescales require full dynamic coupling as implemented here.

The fourth limitation is the one-constant elastic approximation embodied in the gradient term $\frac{L}{2}\left(\partial_{k}Q_{ij}\right)\left(\partial_{k}Q_{ij}\right)$. This approximation assumes that the splay, twist, and bend elastic constants are equal, which is a simplification of the full Frank elasticity that characterizes real nematic and smectic liquid crystals [22]. While anisotropic elastic constants can be incorporated by replacing the single coefficient *L* with a tensor of Frank constants, the present formulation has not implemented this generalization. The one-constant approximation may be inadequate for systems where the anisotropic elastic response is essential for the phase behavior, such as in bent-core liquid crystals or systems with strong chiral interactions.

The fifth limitation concerns the treatment of fluctuations. The noise term in Equation (17) is modeled as Gaussian white noise with an effective temperature $T_{\mathrm{eff}}$. While this captures the leading-order effects of non-equilibrium fluctuations in the driven medium, it is an approximation that may fail to capture the highly directional, colored noise spectra encountered in strongly turbulent active regimes. The fluctuation-dissipation relation is enforced locally, but the effective temperature may be spatially and temporally varying in inhomogeneous active systems. A more complete treatment would require a full stochastic hydrodynamics formulation with multiplicative colored noise and spatially dependent fluctuation-dissipation relations.

The sixth limitation is the absence of an independent energy balance equation in the present formulation. The framework describes the structural evolution of the medium but does not track the localized heat generation, the rate-dependent microscopic work input, or the total structural dissipation rates. This means that the framework is not thermodynamically closed in the sense of a complete energy balance. It relies on the assumption that the energy injected by active driving is parameterized by the active torque number and the effective temperature. For systems where thermal effects, local heating, or heat transport play a significant role, an energy equation would need to be appended to the complete coupled system.

The seventh limitation is the assumption of incompressibility for the flow field *U*, in Equation (22c). While this is appropriate for dense fluid systems and for the colloidal monolayers investigated here, it is not valid for compressible active systems such as bacterial suspensions with significant density variations or gas-like active matter. The framework can be extended to compressible flows by relaxing the incompressibility constraint and adding an equation of state, but this generalization has not been implemented.

The eighth limitation, closely related to the phenomenological nature of the free energy expansion, is that the functional geometry of the nonlinear dissipation potential and several bulk phenomenological coefficients must still be obtained empirically for each specific material system under investigation. While the framework provides a systematic and thermodynamically consistent structure, the material-specific parameters must be determined from experimental data or from more microscopic theories for each system. This empirical parameterization is a standard feature of phenomenological formulations, but it limits the predictive power of the framework in the absence of prior experimental characterization.

Despite these limitations, the framework provides a comprehensive and unified methodology for analyzing sequential symmetry-breaking transitions in driven, far-from-equilibrium complex fluids.

## 6. Conclusions

This study has presented a unified, non-equilibrium phenomenological framework that characterizes the spontaneous emergence of macroscopic order in driven, far-from-equilibrium complex fluids and soft matter systems. The framework is built upon a hierarchical set of postulates that combine phenomenological flexibility with thermodynamic rigor. At its foundation lies the choice of a single symmetric, traceless second-rank tensor $Q\left(r,t\right)$ as the fundamental structural descriptor, which generalizes the classical nematic director description to accommodate biaxiality and topological defects while providing a self-consistent description of orientational anisotropy independent of specific microscopic constituents. This tensorial order parameter is complemented by two auxiliary fields: a conserved scalar field $\phi \left(r,t\right)$ that acts as a generalized thermodynamic control parameter mediating the hierarchical instability thresholds, and an emergent polar vector field $P\left(r,t\right)$ that captures dipolar order arising from the tensorial scaffold via a secondary bifurcation.

The framework is thermodynamically constrained through the introduction of a convex dissipation potential $\Psi \left(\dot{Q}\right)$ that establishes the general nonlinear flux–force relation $H_{\mathrm{total}}=\partial \Psi /\partial \dot{Q}$, guaranteeing non-negative entropy production $\dot{s}=H_{\mathrm{total}}:\dot{Q}\geq 0$ as a consequence of the second law of thermodynamics. This thermodynamic consistency is maintained even when the specific phenomenological closure is varied, provided the mobility coefficients remain positive-definite and the dissipation potential remains convex along accessible trajectories. The departure from standard linear Onsager relations through the state-dependent jamming mobility $\Gamma \left(S\right)=\Gamma_{0}\left(1-S^{2}/S_{\max}^{2}\right)$ represents a key advancement of the formulation, capturing the saturation of structural relaxation and localized kinetic arrest in densely packed, highly ordered active systems.

The free energy landscape, encoded in the generalized Landau–de Gennes expansion with sixth-order penalties and an exponentially decaying tensor-polar coupling coefficient $\gamma \left(S\right)=\gamma_{0}e^{-\lambda S}$, embodies the hierarchical competition between orientational ordering, dipolar alignment, packing constraints, and spatial gradients. The hierarchical modulation of the instability thresholds through the scalar field ϕ ensures that the system traverses a sequential symmetry-breaking cascade (isotropic to nematic to polar) as the control parameter is varied by cooling or increasing packing density. This sequential progression, driven by the feedback between the tensorial scaffold and the emergent polar field, captures the essential physics of the experimentally observed phase sequences in both polar liquid crystals [18] and Janus colloids [19].

The dimensionless parameterization of the complete coupled system compresses the far-from-equilibrium state space into a minimal set of independent dimensionless groups. The active torque number ϵ emerges as a universal bifurcation parameter that governs the onset of spontaneous ordering across all investigated regimes. Linear stability analysis of the homogeneous steady state reveals the universal threshold condition $\epsilon >\epsilon_{c}≃1$, below which thermodynamic restoring forces dominate and the system remains quiescent, and above which active driving overpowers localized dissipation and induces spontaneous macroscopic ordering and spatial pattern formation. This threshold condition, which applies across both static Turing-type instabilities and true dynamical bifurcations, shows that the transition from isotropic disorder to coherent macroscopic patterns in driven media is determined by a common thermodynamic-kinematic balance operating independently of specific microscopic interactions.

The numerical implementation of the complete coupled system, employing staggered-grid finite differences with semi-implicit Crank–Nicolson schemes for stiff diffusion terms and operator-splitting for the field coupling, has enabled quantitative comparisons with two distinct experimental systems (polar liquid crystals [18] and Janus colloids [19]).The predicted phase boundaries agree with experimental observations across length scales ranging from nanometers to micrometers and for interaction mechanisms ranging from dipolar alignment to purely repulsive turn-away torques. This agreement supports the generality of the framework and identifies the active torque number as a common control parameter for structural bifurcations in the regimes examined.

The limitations of the present formulation define clear pathways for future development. The phenomenological free energy expansion, the one-constant elastic approximation, the slaving approximation for the polar field, the effective temperature treatment of fluctuations, and the absence of an independent energy balance equation represent specific assumptions that may require generalization for systems beyond those investigated here. Nevertheless, the framework’s modular architecture—characterized by the separation of phenomenological inputs from thermodynamic constraints, an objective kinematic formulation, dimensionless parameterization, and the identification of candidate universal bifurcation parameters—provides a foundation for such extensions. 

## Figures and Tables

**Table 1:** Dimensionless parameters and their correspondence to experimental observables for the polar liquid crystal system [18] and the Janus colloid system [19].

Parameter	Liquid Crystal	Janus Colloid	Experimental Correspondence
Scalar control field $\left(\phi^{*}\right)$	T-dependent	Area fraction	Temperature/packing density
Active torque number (ϵ)	$\alpha E^{2}/\left(A_{0}Q_{0}\right)$	$\alpha v_{0}^{2}/\left(A_{0}Q_{0}\right)$	Electric field/propulsion speed
Confinement parameter (σ)	∼50	∼10	System size/correlation length
Tensor-polar coupling ${(g}_{0})$	∼1	∼0.5	Dipolar-nematic interaction strength
Coupling decay constant (λ)	∼2	∼1	Steric hindrance attenuation
Cubic coefficient (b)	0.3	0	First-order transition strength
Sixth-order tensor coefficient (e)	0.1	0.2	Steric packing constraint
Sixth-order polar coefficient ${(e}_{P})$	0.1	0.2	Polar saturation penalty
Deborah number (De)	≪1	∼1	Cooling rate/flow relaxation

## Data Availability

Data available upon reasonable request from the authors.
